# Optimized CoQ10 delivery *via* ZnO/CuO nanoparticles: greater *in vitro* cell cycle arrest and *in vivo* tumor suppression than unformulated coenzyme Q10

**DOI:** 10.1039/d6ra02842j

**Published:** 2026-07-21

**Authors:** Lamiaa A. A. Barakat, Radwa K. A. Hassan, Salma M. Khirallah, Mohammed El Behery, Manar A. El-Zend

**Affiliations:** a Chemistry Department(Biochemistry Division), Faculty of Science, Port Said University Port Said 42526 Egypt m_elzend@yahoo.com; b Faculty of Applied Health Sciences, East Port Said National University Port Said Egypt

## Abstract

Coenzyme Q10 (CoQ10) displays significant anticancer effects, but its potential medical application suffers from its inability to dissolve in water and low rates of absorption. CoQ10-loaded zinc oxide (ZnO-Q10NPs) and copper oxide (CuO-Q10NPs) nanocomposites were developed through co-precipitation synthesis, which used CoQ10 as both a capping and stabilizing agent. FTIR XRD, SEM/EDX, TEM, and XPS were used to confirm that the particles maintained spherical shapes with particle sizes of approximately 30.5 nm for ZnO-Q10NPs and 15 nm for CuO-Q10NPs. MTT assay against MCF-7, HepG2, and Caco-2 cell lines revealed that ZnO-Q10NPs exhibited the highest potency against MCF-7 breast cancer cells (IC_50_: 3.65 µg mL^−1^), outperforming free CoQ10, CuO-Q10NPs, and standard chemotherapeutics, while CuO-Q10NPs showed preferential activity against HepG2 cells (IC_50_: 9.46 µg mL^−1^). ZnO-Q10NPs caused MCF-7 cells to enter G_0_/G_1_ arrest at 73.7% while also causing both early and late apoptosis together with Bax and Caspase-3 upregulation and Bcl-2 downregulation, and topoisomerase IIβ and NRF-2 and TNF-α and β-catenin pathways. The two formulations achieved hepatorenal biomarker normalization together with antioxidant enzyme activity restoration and IL-6-mediated inflammation reduction. The results demonstrate that ZnO-Q10NPs function as a strong multitargeted nanotherapeutic option for breast cancer, which needs additional clinical evaluation.

## Abstract 2

Simple schematic diagram of the research process in this study.

## Graphical abstract

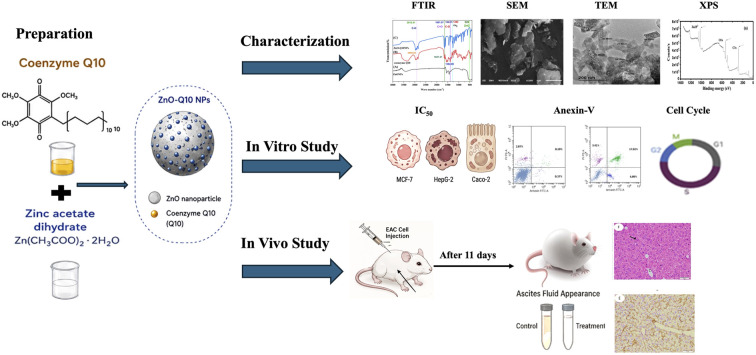

## 1. Introduction

Breast cancer (BC) ranks among the leading causes of mortality worldwide, with projections indicating a substantial increase in incidence among women by 2030. While early-stage disease responds reasonably well to conventional modalities, including surgery, chemotherapy, and radiotherapy, advanced breast cancer presents formidable therapeutic challenges that necessitate innovative strategies.^1^ Chemotherapy remains a cornerstone of systemic treatment; however, its efficacy is frequently compromised by indiscriminate cytotoxicity toward non-malignant cells.^2^

The high propensity for relapse in mice, severe adverse effects, and acquired multidrug resistance underscore the limitations of current therapeutic paradigms. Consequently, natural products have emerged as compelling alternatives in breast cancer management, demonstrating potent antiproliferative and anticancer activities alongside substantial therapeutic potential. Plant-derived natural products, in particular, have garnered considerable attention for their efficacy across various malignancies. These agents primarily suppress breast cancer progression by inducing apoptosis and inhibiting metastatic dissemination, thereby addressing key hallmarks of oncogenesis.^3^

Natural products have historically played a pivotal role in oncology, forming the basis for 60% of modern chemotherapeutic agents and providing diverse lead compounds.^4^ In BC, phytochemicals and endogenous compounds modulate key hallmarks of malignancy, dysregulated proliferation, apoptosis evasion, angiogenesis, chronic inflammation, oxidative stress, and metastasis with lower systemic toxicity than synthetic alternatives. Many exhibit chemopreventive and chemo sensitizing effects, enhancing standard therapies and mitigating adverse outcomes, making them ideal for integrated or adjuvant regimens.^3,5,6^

Coenzyme Q10 (CoQ10) plays critical roles in cellular functions, including energy production, redox balance, and metabolic processes. Various micronutrients prevent free radical formation associated with carcinogenesis, and CoQ10 is prominent among them. CoQ10's ubiquitous distribution reflects its physiological necessity, with the highest concentrations in metabolically active organs such as the heart, kidney, and liver.^7^ Also known as ubiquinone-10, this lipophilic benzoquinone derivative, naturally present in human mitochondria and dietary sources like organ meats, fatty fish, and nuts, has gained significant attention due to its multifaceted biological functions, including potent antioxidant activity, membrane stabilization, and bioenergetic support. Studies demonstrate CoQ10's anticancer properties in BC through reactive oxygen species (ROS) scavenging, suppression of chronic inflammation, induction of apoptosis *via* both intrinsic mitochondrial and extrinsic pathways, and inhibition of tumor growth and metastatic spread. It enhances BC cell sensitivity to conventional chemotherapy while protecting normal tissues.^8^

However, CoQ10's extremely poor water solubility, high molecular weight, and limited gastrointestinal absorption severely restrict its bioavailability and therapeutic utility, necessitating advanced nanoformulation strategies.^9^ Beyond their direct molecular effects, nanoparticle-based delivery systems address these limitations by enhancing solubility through surface adsorption or conjugation, improving cellular uptake *via* endocytosis, enabling tumor-specific accumulation through the enhanced permeability and retention (EPR) effect, and providing stimulus-responsive release in the tumor's acidic and oxidative microenvironment.^10^ In these systems, CoQ10 functions as both a stabilizing agent during nanoparticle synthesis and a chemosensitizer that amplifies therapeutic efficacy while reducing required dosages and systemic toxicity.^11^

Current approaches leverage nanotechnology to maximize CoQ10's potential in BC therapy. Zinc oxide (ZnO) and copper oxide (CuO) nanoparticles possess unique physicochemical properties and intrinsic anticancer capabilities through tumor-selective ROS modulation, inducing mitochondrial dysfunction, DNA damage, and apoptosis in BC cells while minimizing harm to normal tissues.^12^ Surface modification with CoQ10 creates hybrid nanomedicines that synergistically combine the inorganic core's cytotoxicity with CoQ10's anti-inflammatory and pro-apoptotic benefits.^13^ Synthesis of ZnO and CuO nanoparticles using CoQ10 as a reducing, stabilizing, and capping agent represents a particularly promising green strategy. ZnO- and CuO-Q10 nanostructures exhibit markedly improved water solubility, cellular uptake, and physiological stability compared to free CoQ10. In BC models, their combined action drives enhanced apoptosis through multiple pathways, robust proliferation inhibition, suppression of chronic inflammation, and reduced angiogenesis and metastasis.^14,15^

The therapeutic advantage of these CoQ10-based nanostructures is in their capacity to coordinate a multi-target molecular response. In particular, they efficiently change the apoptotic threshold by lowering the levels of the anti-apoptotic Bcl-2 and raising the levels of Bax at the same time. This activates Caspase-3.^16^ These hybrid nanomedicines also have strong anti-inflammatory effects because they stop the TNF-α/IL-1β/IL-6 cytokine cascade.^10^ They enhance cellular cytoprotection through the Nrf-2 pathway and disrupt oncogenic signaling pathways, including the Wnt/β-catenin pathway and topoisomerase activity, which are often dysregulated in aggressive breast cancer morphologies.^17^

ZnO-Q10NPs excel due to the pH-responsive properties of ZnO dissolution and selective ROS generation in the mildly acidic tumor microenvironment (pH 6.5), conferring targeted cytotoxicity toward malignant cells while sparing normal tissues. Surface-bound CoQ10 further optimizes this selectivity by attenuating oxidative damage in healthy cells while potentiating apoptosis in dysregulated BC cells. Likewise, CuO-Q10 nanoparticles synergize the pro-oxidant effects of CuO with CoQ10's inflammation-modulating and apoptosis-inducing properties, achieving an optimal balance between tumor eradication and normal tissue preservation.^18^ Thus, ZnO-Q10 and CuO-Q10 nanoparticles emerge as innovative therapeutic platforms that overcome CoQ10's solubility barriers while synergistically combining its multitargeted anti-inflammatory and pro-apoptotic bioactivity with metal oxide nanoparticles' intrinsic antitumor mechanisms, addressing breast cancer's key therapeutic challenges with promises of reduced resistance, minimized toxicity, and enhanced patient survival outcomes. Based on this potential, the present study examined the efficacy of ZnO-Q10 and CuO-Q10 nanoparticles as innovative breast cancer therapies in all areas. This study seeks to examine the influence of the inherent anticancer properties of metal oxide cores and the enhanced bioactivity of nano-formulated CoQ10 on the Nrf-2/TNF-α signaling pathway, the modulation of β-catenin and topoisomerase activities, and the equilibrium between Bax and Bcl-2 in apoptosis. The major purpose of this study is to prove that these hybrid nanostructures are good medications that can be used in many different settings without producing difficulties with systemic toxicity and solubility. This could be a smart method to assist individuals with advanced breast cancer to live longer and be healthier.

### 1.1. Chemistry

#### 1.1.1. Chemicals

All reagents employed in this study were of excellent purity (98–99%). We procured zinc, copper(ii) acetate monohydrate, and coenzyme Q10 extract from Sigma-Aldrich. We acquired NaOH from CDH in India.

#### 1.1.2. Phyto synthesis of ZnO and CuO nanoparticles

ZnO nanoparticles were co-precipitated using NaOH. Zinc acetate (Zn (CH_3_COO)_2_·2H_2_O, 2.2 g) and NaOH (0.8 g) were dissolved in 50 mL of D.W. Zinc acetate was gradually added to NaOH while magnetically stirring for six hours. Add 20 mg mL^−1^ of COQ10 incorporated into the mixture using EtOH and stir for 6 h. After 5 days of desiccation at 50 °C, the product was centrifuged at 10 000 rpm to make powder. The same process was used to produce CuO nanoparticles, substituting zinc acetate with copper (Cu(CH_3_COO)_2_).^19^

#### 1.1.3. Characterization of the nanoparticles

CoQ10 and the synthesized nanoparticles were analyzed by SEM (JEOL JSM-6510LV) with integrated EDS. Particle shape and size distribution were evaluated using TEM (JEM-1400 Plus). FTIR spectra were recorded in the 4000–400 cm^−1^ range. XRD patterns were collected on a Siemens D-500 diffractometer with Cu-Kα radiation (*λ* = 0.15418 nm) at 2*θ* = 2–70°. XPS analysis was performed using an ESCALAB 250Xi system (Thermo Fisher-VG Scientific).

### 1.2. Biological methods

#### 1.2.1. *In vitro* study

##### 1.2.1.1. *In vitro* cytotoxicity study against MCF-7, HepG-2 and Caco-2 cancer cell lines

The human cancer cell lines (MCF-7 breast adenocarcinoma, HepG2 hepatocellular carcinoma, and Caco2 colorectal adenocarcinoma) in this study were obtained from the Holding Company for Biological Products and Vaccines (VACSERA), Cairo, Egypt. The synthesized compounds were evaluated for *in vitro* cytotoxicity against three human cancer cell lines: breast, hepatocellular, and colorectal adenocarcinoma, using the MTT cell viability assay. Cells were seeded at 1 × 10^4^ cells per well in flat-bottomed culture plates using complete growth media, then pre-incubated for twenty-four hours at 37 °C to ensure proper monolayer formation. After 24 h, cells were exposed to graded serial concentrations of each test drug (dissolved in DMSO) for 48 h. The DMSO concentration was maintained below 0.5% (v/v) in all experimental and control wells to prevent solvent interference with cell viability. Doxorubicin served as the positive control in all assays. Cell viability and IC_50_ values were determined using the MTT assay. Experiments were conducted in triplicate.^20^

##### 1.2.1.2. Flow cytometric analysis of ZnO-Q10NPs treated MCF-7 cells for cell cycle progression

DNA content was analyzed by PI staining to determine cell cycle distribution. MCF-7 cells (2 × 10^5^ cells per well) were seeded overnight at 37 °C in 5% CO_2_, then treated with ZnO-Q10NPs at their IC_50_ concentration (3.64 µg mL^−1^) for 24 h. Following treatment, cells were harvested, fixed in cold 70% EtOH, and stained with PI supplemented with RNase. Flow cytometry was performed to acquire ≥10 000 events per sample, and the data were used to determine the % of cells in G_0_/G_1_, S, and G_2_/M phases.^21^

##### 1.2.1.3. Flow cytometric analysis of apoptosis in ZnO-Q10NPs treated MCF-7 cells by annexin V-FITC/PI double staining

Apoptotic cell death was quantified by flow cytometry using an Annexin V-FITC/Propidium Iodide (PI) apoptosis detection kit (Bio-Vision, Milpitas, CA, USA) according to the manufacturer's instructions. MCF-7 cells were seeded at 2 × 10^5^ per well and treated with ZnO-Q10NPs at IC_50_ (3.64 µg mL^−1^) for 24 h. Following treatment, cells were gently trypsinized, centrifuged, and washed twice with ice-cold PBS to remove culture medium. The binding buffer was used to resuspend cell pellets at the right concentration. Cells were co-incubated with Annexin V-FITC and PI staining solution for 45 min at room temperature in the dark to detect early and late apoptosis. The study used FITC channel detectors to identify viable, early, late, and necrotic cells using flow cytometry.^22^

##### 1.2.1.4. Gene expression profiles of ZnO-Q10NPs treated MCF-7 cells by quantitative RT-PCR analysis

An RT-qPCR analysis. Adhering to the manufacturer's guidelines, the RNeasy Mini Kit (Qiagen, Hilden, Germany) retrieved total RNA from MCF-7 cells. Cells were treated with ZnO-Q10NPs at 3.64 µg mL^−1^ (IC_50_), and untreated cells were used as negative controls. To synthesize cDNA from extracted RNA, the RT^2^ First Strand Kit was used. The Rotor-Gene 6000 thermocycler (Qiagen Corbett, Sydney, Australia) was used for quantitative real-time PCR (qRT-PCR) using RT^2^ SYBR® Green ROX™ FAST master mix and gene-specific primer pairs. GAPDH was the internal reference gene. The thermal cycling technique included enzyme activation at ninety-five degrees Celsius for ten minutes, 40 cycles of amplification at ninety-five degrees Celsius for fifteen seconds, and annealing/extension at 60 °C for 30 s. Melting curve analysis verified primer specificity and the absence of non-specific amplification products after each run. Fold-change values were obtained using the 2^−^ΔΔ*C*_t_ technique. Table S1 displays primer sequences for BAX, Caspase-3, IL-1β, β-catenin, TOPO II, and BCL-2 quantification. All trials were done in triplicate, and data are shown as average ± SD.^12^

##### 1.2.1.5. Quantification of NRF-2 expression levels in ZnO-Q10NPs treated MCF-7 cells by sandwich ELISA

The intracellular levels of NRF-2 protein in MCF-7 cells treated with ZnO-Q10NPs at IC_50_ (3.64 µg mL^−1^) were evaluated using a ELISA according to following instructions.^23^ Negative controls were untreated cells. Briefly, standards and cell lysate samples were added to the microplate wells precoated with an NRF-2-specific monoclonal capture antibody and incubated for 2.5 h at room temperature with gentle agitation to facilitate antigen–antibody interaction. After the incubation interval the wells were extensively washed to remove unbound material, and a biotinylated anti-NRF-2 detection antibody was added. Then, streptavidin- HRP conjugate was added to each well and incubated for 45 min to form a detectable immunocomplex. Following another wash step, the TMB chromogenic substrate was added and allowed to develop color for ∼30 min at RT in the dark. The stop solution was added, and the absorbance was read at 450 nm in a microplate reader. Unknowns were extrapolated using a standard calibration curve created from serial dilutions of the specified standards. All incubation steps were performed with gentle shaking, and stringent washing protocols were adopted during the course of the experiment to ensure the specificity and sensitivity of the test. All samples were run in triplicate, and findings are reported as mean ± standard deviation.

##### 1.2.1.6. Western blot method

Protein concentrations of TNF-α, β-catenin, and β-actin in MCF-7 cells treated with ZnO-Q10NPs and untreated controls were assessed using western blotting. Cells were lysed in RIPA buffer containing protease inhibitors, and protein quantities were quantified using the BCA assay. Equal quantities of protein (20–50 µg) were denatured, subjected to SDS-PAGE, and subsequently transferred to PVDF membranes (Bio-Rad Laboratories, Hercules, CA, USA). Membranes were obstructed and incubated with primary antibodies, TNF-α (Cat. No. #3707; Cell Signaling Technology, Danvers, MA, USA), β-catenin (Cat. No. #2698; Cell Signaling Technology, Danvers, MA, USA), and β-actin (Cat. No. A5441; Sigma-Aldrich, St. Louis, MO, USA), subsequently followed by HRP-conjugated anti-mouse IgG secondary antibody (Cat. No. #7076; Cell Signaling Technology, Danvers, MA, USA). Bands were detected by ECL and photographed with a Chemi Doc. Densitometric analysis was conducted using ImageJ and normalized to β-actin.^24^

#### 1.2.2. *In vivo* study

##### 1.2.2.1 Experimental animals and ethical approval

Abou Rawash's Giza Society provided us with an adult Swiss albino female mouse that weighed twenty to twenty-five grams. During the week leading up to the experiment, the mice were given free access to food and water at the Animal House at Port Said University's Faculty of Science. This allowed them to adjust to their new surroundings. Scientific Investigation Ethics Committee of Port Said University gave their approval to the (ERN: PSU.Sci.96).

##### 1.2.2.2. Neoplastic specimen

Getting EAC tumor cells from Egypt's National Cancer Institute in Cairo was the first step in the transplant process. Placed in the belly of Swiss albino female mouse and given ascites intraperitoneally every 8 to 10 days, the animals were kept alive.

##### 1.2.2.3. Acute toxicity

The acute toxicity of ZnO-Q10NPs and CuO-Q10NPs was determined by the approach of Meier and Theakston. LD_50_ values were calculated by dosing mice (*n* = 4 animals per dosage group) with graduated doses of the test substances (1–10 mg kg^−1^). Another series of groups of 5 mice each was given graduated doses of the test substances (20–100 mg kg^−1^). The mice were watched for a day post injection.^25^

##### 1.2.2.4. Effect of treatments on cell count and ascites tumor volume of EAC

Female Swiss albino mice (20–25 g) were randomly divided into eleven experimental groups (*n* = 6 per group) consisting of negative control, positive control (EAC) and 9 treatment groups. Day 1, mice were infected intraperitoneally with 2.5 × 10^6^ viable EAC cells per mouse in positive control and treatment groups. The treatment groups were given free coenzyme Q10 (CoQ10), ZnO-Q10NPs, and CuO-Q10NPs in doses of 5, 10 or 20 mg kg^−1^ body weight, given intraperitoneally as per the experimental protocol. All treatments were administered intraperitoneally on alternate days for a total of five doses. On day 11, all mice were anesthetized according to the approved institutional animal care protocol,^26^ and the ascitic fluid was aseptically aspirated from the peritoneal cavity using a sterile syringe to measure the ascitic tumor volume and count. Following sample collection, the mice were euthanized in accordance with the approved institutional ethical guidelines.^27^ The ascitic fluid was diluted with heparinized normal saline and combined with trypan blue solution for viable cell counting by standard trypan blue exclusion method.^28^ The cell suspension was incubated for 2 min at room temperature (25 °C) and then counted using a Thoma hemocytometer at a magnification of ×40. Live cells did not take up the dye and remained unstained, but dead cells were stained blue. Viable EAC cell count was given as cells per mL. Blood samples were taken for serum separation. The liver and kidney tissues were removed and separated into two halves. One part was fixed in 10% neutral buffered formalin for histopathological and immunohistochemical investigations, while the other part was immediately kept at −80 °C for biochemical and molecular analysis. Data were presented as mean ± SD and analyzed with GraphPad Prism software. Statistical significance was defined as *P* < 0.05.

##### 1.2.2.5. Biochemical tests

Serum indicators of hepatic functions, including alanine aminotransferase (ALT) and aspartate aminotransferase (AST), were determined using commercial enzymatic assay kits from TECO Diagnostics (Anaheim, CA, USA),^29^ while renal functions, including creatinine (Cat. No. DICT-500) and urea (Cat. No. DIUR-500), were evaluated using commercial kits from BioAssay Systems (Hayward, CA, USA) according to the manufacturers' procedures,^30^ and the findings were represented in standard units.

##### 1.2.2.6. Biochemical investigation of oxidants and antioxidants

Relevant tissue homogenates were analyzed for oxidative stress and antioxidant parameters using commercially approved assays. Catalase (CAT) activity was assessed using a MyBioSource kit (San Diego, CA, USA; Cat. No. MBS006963), lipid peroxidation was measured using a MyBioSource kit (Cat. No. MBS268427),^31^ and superoxide dismutase (SOD) activity was determined using a MyBioSource kit (Cat. No. MBS036924). Additionally, reduced glutathione (GSH) levels were evaluated using a Shanghai BlueGene Biotech Co., Ltd kit (Shanghai, China; Cat. No. E02G0367) according to the manufacturer's procedures, and findings were represented in units provided by the kit.^32^

##### 1.2.2.7. Histopathological examination

Hepatic and renal tissues were fixed in 10% neutral-buffered formalin, sectioned, and stained with hematoxylin and eosin (H&E), following standard tissue processing and H&E staining protocols. Renal sections were evaluated semi-quantitatively for indicators of acute tubular damage, including necrosis, apical vacuolization with accompanying brush border loss, and epithelial flattening. Hepatocellular injury was assessed with a modified histological grading system based on established criteria.^33^

##### 1.2.2.8. Immunohistochemistry examination

Immunohistochemistry for IL-6 was conducted on paraffin sections following antigen retrieval and inhibition of endogenous peroxidase and protein binding. Sections were treated with anti-IL-6 primary antibody (Cat. No. ab290735; Abcam, Cambridge, UK) and suitable HRP-conjugated secondary antibody Cat. No. ab205718; Abcam, Cambridge, UK), followed by chromogenic development and counterstaining (typical IHC procedure). IL-6 expression was measured semi-quantitatively by staining intensity and percentage of positive cells; digital image analysis was used when quantitative measurement was needed.^34^

##### 1.2.2.9. Statistical analysis

Statistical evaluation was conducted with GraphPad Prism version 10.5. Values are expressed as average ± SD. Comparisons between various groups were performed using one-way ANOVA. The interaction between independent variables was assessed using two-way ANOVA. Statistical significance was assessed as p* ≤ 0.05, p** ≤ 0.01, p*** < 0.001, p**** ≤ 0.0001.

## 2. Results and discussion

### 2.1. Chemistry

#### 2.1.1. FTIR characterization of coenzyme metal oxide nanocomposites

**Fig. 1:**
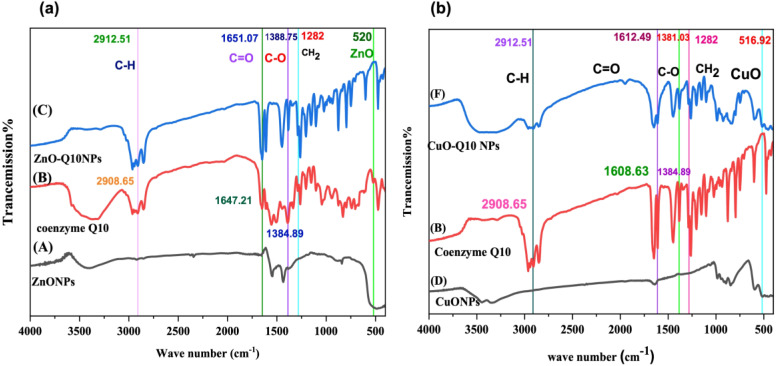
FTIR spectra of (a) ZnO-Q10NPs and (b) CuO-Q10NPs.

**Fig. 2:**
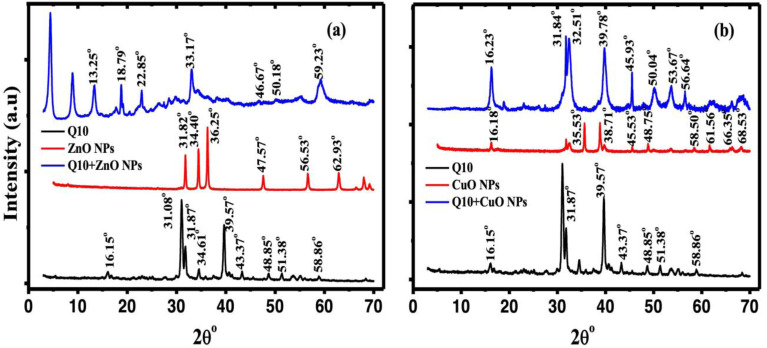
XRD patterns of (a) ZnO-Q10NPs and (b) CuO-Q10NPs.

FTIR spectra were performed to understand the chemical and structural nature of coenzyme Q10 coated with ZnO and CuO nanoparticles. Fig. 1 illustrates the FTIR spectrum of the CoQ10 coated with ZnO and CuO nanoparticles. The FT-IR spectra confirmed the interaction between the metal oxide and CoQ10 in the wavenumber range of 400–4000 cm^−1^. The characteristic FT-IR spectrum of the CoQ10 blend is depicted in Fig. 2(b). CoQ10 shows a band at 2908.65 cm^−1^ that is centered due to the C–H-stretching modes, 2934 cm^−1^ (asymmetric stretching of CH_2_), 1647.21 cm^−1^ due to C=C, and 1384.89 cm^−1^ C–O stretching. Fig. 1(a and c) showed the FT-IR spectra of the ZnO-Q10NPs. It was found that ZnO-NPs had a significant effect on some of the functional groups of the CoQ10 due to the complexation or interactions between these groups and ZnO. The doping level of ZnO-NPs on the CoQ10 films shows little or no change in the FT-IR peaks obtained. These FT-IR findings can be attributed to the formation of secondary bond forces between the CoQ10 and the ZnO-NPs. The FT-IR peaks of the ZnO-doped blend CoQ10 exhibit 2912.51 cm^−1^ for C–H stretching modes, 1651.07 cm^−1^for C=C, and 1388.75 cm^−1^ for C–O. These FT-IR results for the ZnO-Q10NPs are consistent with the structural properties of Zn^2+^ ions doped into CoQ10 NPs. The peak at 520 cm^−1^ indicated Zn–O.

Fig. 1(b and f) show the FT-IR spectra of the CuO-Q10NPs. It was found that CuO-NPs had a significant effect on some of the functional groups of the CoQ10 due to the complexation or interactions between these groups and CuO. 2912.51 cm^−1^ for C–H 1612.49 cm^−1^ attributed to (C=O), 1381.03 cm^−1^, supporting the coordination of C–O to CuO. The new band at 516.92, 455.20 cm^−1^ indicated the Cu–O stretching vibration.

#### 2.1.2. XRD characterization and crystallite size of coenzymeQ10, ZnO, CuO, and their nanocomposites

Fig. 2(a) shows the X-ray diffraction (XRD) patterns of pure CoQ10, ZnO-NPs, and ZnO-Q10NPs in the 2*θ* range from 5° to 70° for investigating the crystal structure of the samples. The XRD pattern of pure CoQ10 displays numerous sharp diffraction peaks throughout the scan range, indicating its high crystalline nature, with characteristic reflections observed at approximately 2*θ* = 16.15°, 31.08°, 34.61°, 39.57°, 43.37°, 48.85°, 51.38°, and 58.86°, indicating the highly crystalline nature of CoQ10.^35^ Also, XRD of Zn- NPs Exhibited diffraction peaks at 2*θ* = 31.82°, 34.40°, 36.25°, 47.57°, 56.53°, 62.93°, and 67.9°, corresponding to the (100), (002), (101), (102), (110), (103), and (112) planes of the hexagonal wurtzite phase of ZnO. [JCPDS card No. 36-145].^36^ The sharp, well-defined peaks confirm the high crystallinity of ZnO-NPs. On the other hand, it is found that the diffraction peaks of CoQ10 are shifted and decreased in the XRD of ZnO-Q10NPs. This may be due to the strong interaction between CoQ10 and ZnO. Similarly, Fig. 2(b) illustrates XRD patterns of CoQ10, CuO-NPs, and CuO-Q10NPs recorded in the 2*θ* range from 5° to 70°. The diffraction pattern of pure CuO-NPs shows distinct peaks at approximately 2*θ* = 16.18°, 35.53°, 38.71°, 48.75°, 58.50°, 61.56°, and 66.3° corresponding to the crystal planes of (110), (002), (111), (−202), (202), (−113), (022), confirming its high crystallinity. These planes correspond well to [JCPDS Card No. 01-089-2529].^37^

The X-ray diffraction (XRD) pattern of ZnO-Q10 and CuO-Q10 NPs exhibits characteristics common to rutin, ZnO-NPs, and CuO-NPs. This pattern is characterized by broad, remarkably low-intensity CoQ10 peaks, while the characteristic peaks of both zinc oxide and copper oxide remain visible with slight intensity reductions and minor peak shifts. These changes indicate the successful incorporation and interaction of CoQ10 molecules with ZnO-NPs and CuO-NPs. The decrease in the peak intensity of CoQ10 suggests a partial loss of crystallinity due to the coordination of hydroxyl and carbonyl groups in rutin with positive Zn^2+^ and Cu^2+^ ions, forming a surface–bound complex around the ZnO-NPs and CuO-NPs. This strong interaction supports the formation of a stable nanocomposite system with enhanced structural integration between organic and inorganic components.^38^

The crystallite size of the CoQ10, ZnO-NPs, CuO-NPs, ZnO-Q10NPs, and CuO-Q10NPs is estimated by using Debye–Scherrer's equation:*D* = 0.94 *λ*/*β* cos *θ*where *λ*, *β* and *θ* are the wavelength of X-ray, the full width at half maximum (FWHM) in radians, and the diffraction angle. It is found that the average crystallite size of these materials is in the range of 23.42, 37.72, 46.61, 21.97, and 31.18 nm, respectively.

#### 2.1.3. Morphology analysis

##### 2.1.3.1. Morphological characterization and surface topography of ZnO-Q10 and CuO-Q10 NPs

**Fig. 3:**
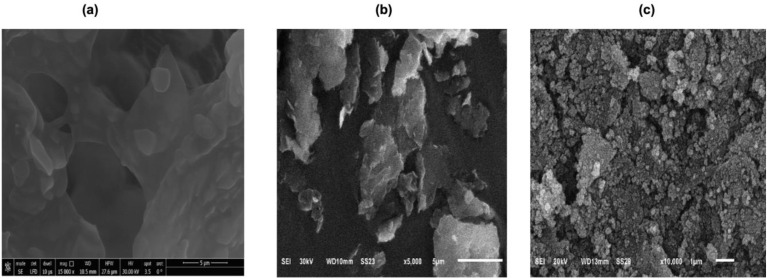
SEM micrographs of (a) CoQ10, (b) ZnO-Q10NPs, and (c) CuO-Q10NPs.

SEM is used to investigate the surface morphology of the CoQ10, ZnO-Q10, and CuO-Q10 samples. SEM of pure CoQ10 typically shows crystalline structures, as can be seen in Fig. 3(a). These structures appear as large, flat slices or as spherical or rod-shaped molecules. On the other hand, Fig. 3(b and c) shows SEM of the ZnO-Q10NPs and CuO-Q10NPs samples. Fig. 3(b) illustrates CoQ10 layers decorated with clusters of zinc oxide nanoparticles distributed over the surface. Meanwhile, Fig. 3(c) shows assemblies in which spherical CoQ10 particles are integrated with copper oxide nanoparticles, indicating successful incorporation of the metal oxide nanoparticles into the CoQ10 matrix.

The roughness of the samples has been investigated, as shown in Fig. S1, and the roughness parameters are estimated and listed in Table (S2). It is found that the CuO-Q10NPs composite is rougher than the ZnO-Q10NPs composite sample. Also, the porosity is estimated from the roughness measurements, as shown in Fig. S2, and it is found that ZnO-Q10NPs is less porous than CuO-Q10NPs, as listed in Table (S2).

##### 2.1.3.2. Elemental composition and distribution analysis by energy dispersive X-ray spectroscopy (EDX)

**Fig. 4:**
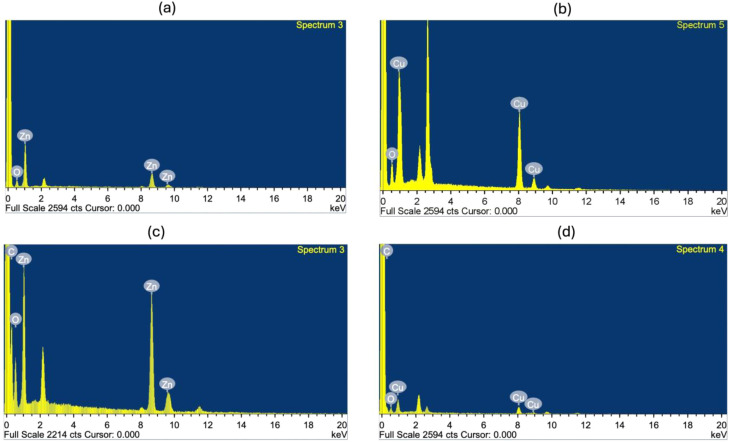
EDX spectrum of (a) ZnO-NPs, (b) CuO-NPs, (c) ZnO-Q10NPs, and (d) CuO-Q10NPs.

Fig. 4 displays EDX spectra of ZnO-NPs, CuO-NPs, ZnO-Q10NPs, and CuO-Q10NPs. The analysis of the EDX spectrum showed the presence of Zn, O, and Cu, as shown in Fig. 4(a and b). This means that Zn, O, and Cu are the main components of the samples, indicating that zinc and copper are in an oxidized state, and no impurities were found in the EDX spectra. On the other hand, the EDX spectrum of ZnO-Q10NPs and CuO-Q10NPs samples showed the presence of Zn, O, Cu, and C, as illustrated in Fig. 6(c and d). The elemental analysis of the nanocomposite components is listed in Tables S3 and S4.

##### 2.1.3.3. Transmission electron microscopy (TEM) analysis of the particle size and morphology of ZnO-Q10NPs and CuO-Q10NPs

**Fig. 5:**
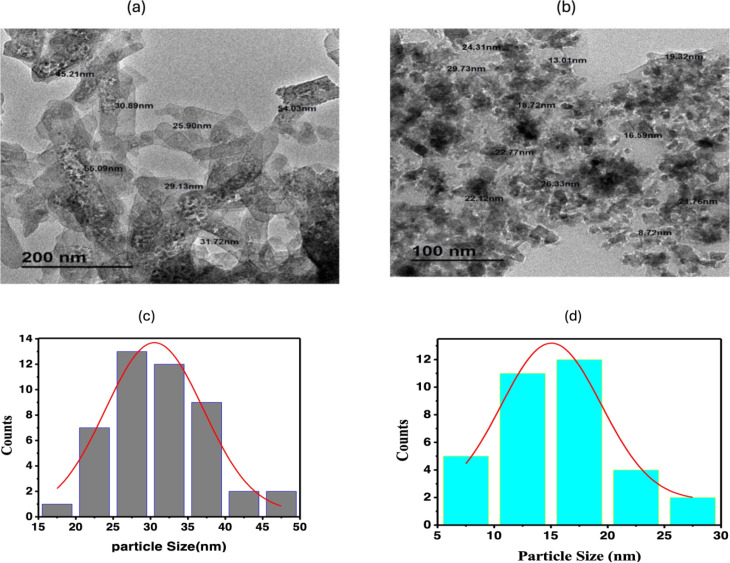
TEM micrographs of (a) ZnO-Q10NPs, (b) CuO-Q10NPs, particle size profile of (c) ZnOQ10NPs, and (d) CuO-Q10NPs.

Transmission electron microscopy (TEM) images of ZnO-Q10NPs and CuO-Q10NPs are shown in Fig. 5(a and b). The TEM image reveals that zinc oxide nanoparticles as spherical/hexagonal nanoparticles in ZnO-Q10NPs, as shown in Fig. 5(a), with an average particle size (approximately 30.47 nm), as shown in Fig. 5(c). Meanwhile, in CuO-Q10NPs, copper oxide nanoparticles exhibited different structures, often clustered in various shapes, such as nanorods and spheres, as depicted in Fig. 5(b), with an average particle size (approximately 14.98 nm), as illustrated in Fig. 5(d).

#### 2.1.4. XPS analysis of surface composition in doped ZnO and CuO nanoparticles

**Fig. 6:**
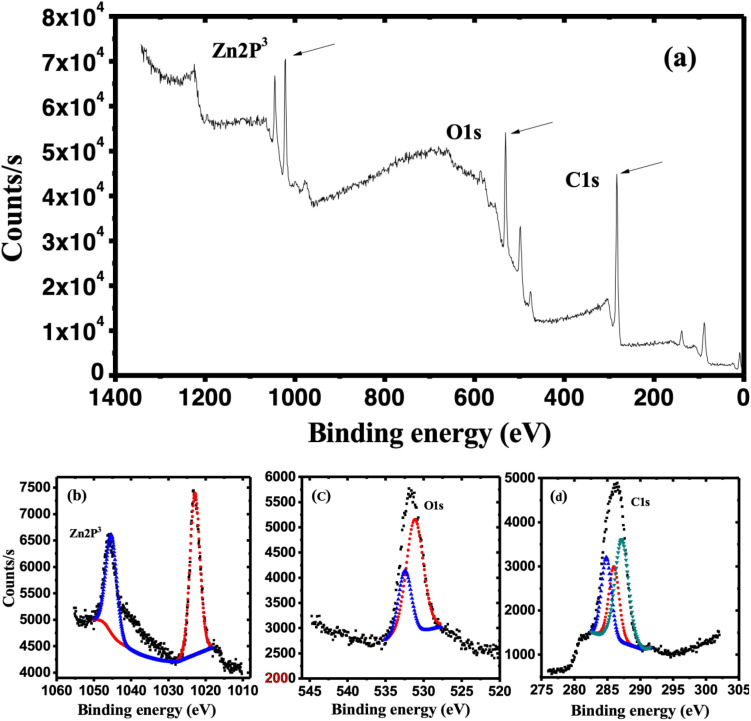
(a) Survey spectra of ZnO-Q10NPs and high resolution of (b) Zn 2p_3_, (c) O 1s, and (d) C 1s.

**Fig. 7:**
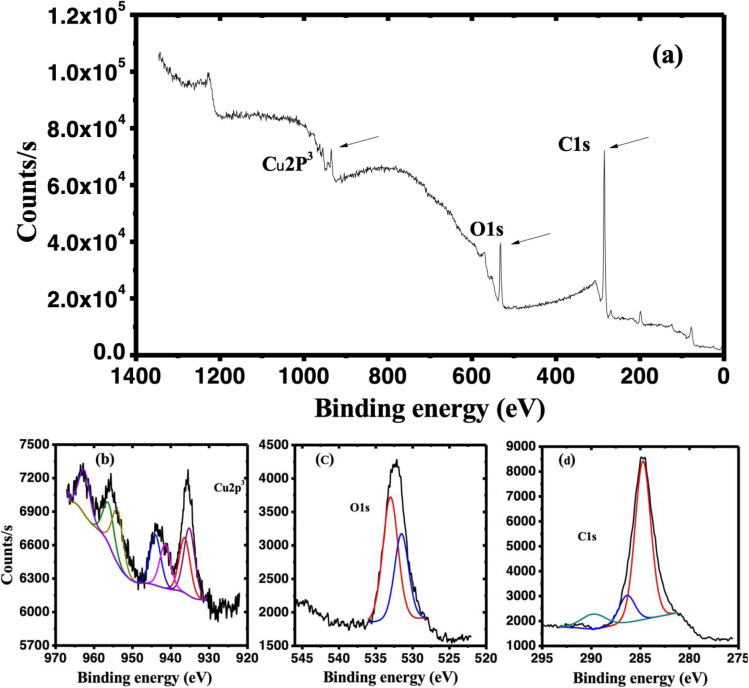
(a) Survey spectra of CuO-Q10NPs and high resolution of (b) Cu 2p_3_, (c) O 1s, and (d) C 1s.

X-ray photoelectron spectroscopy (XPS) was used to investigate the chemical states and composition of the ZnO-Q10NPs and CuO-Q10NPs. The broad XPS survey spectra, Fig. 6(a) and 7(a). Identifying the peaks for O 1s, C 1s, and Zn 2p (or Cu 2p) became a straightforward task. This proved that the ZnO and CuO nanoparticles were made accurately and that adding CoQ10 to them was easy. Fig. 6(b) shows the high-resolution Zn 2p spectra, which display two distinct peaks. The first peak appears between 1022 and 1023 eV, while the second peak is found between 1045 and 1046 eV. The peaks are made up of Zn 2p_3/2_ and Zn 2p_1/2_. Therefore, zinc is in the Zn^2+^ oxidation state, which is the most common form in ZnO nanoparticles. The high-resolution Cu 2p spectra in Fig. 7(b) also showed two clear peaks: one between 933 and 935 eV, and another between 952 and 955 eV. Cu 2p_1/2_ and Cu 2p_1/2_ are these. This means that Cu^2+^ ions are present and that CuO is being produced. The O 1s spectra in Fig. 6 and 7(c) demonstrate that the lattice oxygen in the Zn–O or Cu–O bonds is the most important part. Some portions stick together better than others, such as the carbonyl groups on the surface and the functional groups of CoQ10 that contain oxygen in them. This means that when the metal oxide nanoparticles and CoQ10 molecules interact, they react strongly with each other. The C 1s spectra (Fig. 6 and 7(d)) also exhibit some of the same bonds that are in the poly isoprenoid and aromatic structures of Coenzymeq10, such as the C–C/C=C, C–O, and C=O (or O–C=O) bonds. The XPS results reveal that the ZnO and CuO nanoparticles were manufactured well and that rutin could easily stick to their surfaces since they had functional groups that included oxygen. This makes the surfaces more stable and functional.

### 2.2. Biological activity

#### 2.2.1. *In vitro* studies

##### 2.2.1.1. Evaluation of cytotoxic activity

**Fig. 8:**
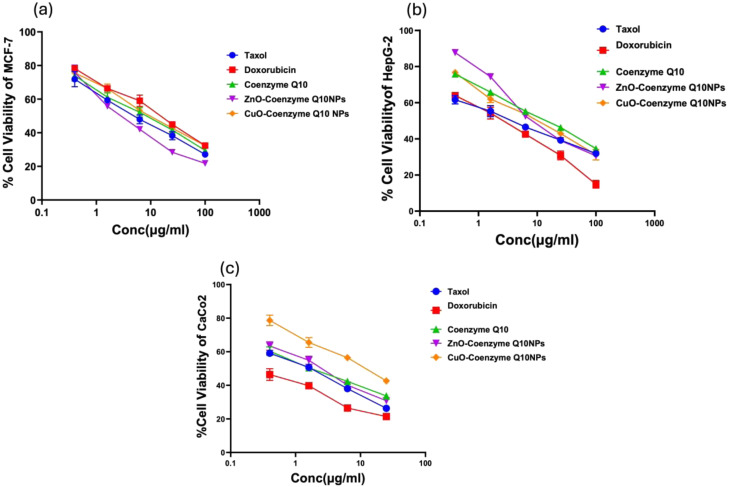
Dose–response curves showing the effect of Co-Q10, ZnO-Q10NPs, CuO-Q10NPs, Taxol, and Doxorubicin on the viability of human cancer cell lines, as determined by the MTT assay. (a) MCF-7 breast cells; (b) HepG-2 cells; (c) Caco2 colorectal cells. Cell viability (%) is plotted against compound concentration (µg mL^−1^). Data are expressed as mean ± SEM of three independent experiments performed in triplicate.

As shown in Fig. 8 and Table (S5), the cytotoxic effects of CoQ10, ZnO-Q10NPs, and CuO-Q10NPs were tested against three human cancer cell lines, which included MCF-7 breast adenocarcinoma, HepG2 hepatocellular carcinoma, and Caco-2 colorectal adenocarcinoma. Doxorubicin and Taxol were used as their standard chemotherapeutic reference drugs. The MCF-7 breast cancer cell line exhibited the highest cytotoxicity with ZnO-Q10NPs, which achieved an IC_50_ of 3.64 ± 0.12 µg mL^−1^ and showed 2.1-fold higher effectiveness than free Co-Q10, which had an IC_50_ of 7.71 ± 0.24 µg mL^−1^. The CuO-Q10NPs showed decreased effectiveness against this cell line with an IC_50_ value of 10.49 ± 0.36 µg mL^−1^. The two reference drugs, Doxorubicin and Taxol, produced IC_50_ results of 13.29 ± 0.41 and 5.42 ± 0.18 µg mL^−1^. The ZnO-Q10NPs treatment achieved better results than both reference drugs in MCF-7 cells, while free CoQ10 produced better results than Doxorubicin. The CuO-Q10NPs showed their strongest effects against HepG2 hepatocellular carcinoma cells when they achieved an IC_50_ value of 9.46 ± 0.28 µg mL^−1^. Doxorubicin and Taxol showed higher effectiveness in this specific cell line because they produced an IC_50_ value of 2.30 ± 0.11 µg mL^−1^ and Taxol 3.52 ± 0.11 µg mL^−1^. The pure CoQ10 showed moderate cytotoxicity with an IC_50_ value of 8.25 ± 0.38 µg mL^−1^, and the ZnO-Q10NPs showed similar effects with an IC_50_ value of 8.68 ± 0.27 µg mL^−1^. The CuO-Q10NPs showed extremely reduced effectiveness against this cell line because their IC_50_ value reached 46.18 ± 1.36 µg mL^−1^, which made them five times less effective than pure CoQ10. The reference drugs demonstrated strong effectiveness because Doxorubicin produced the lowest IC_50_ result during all combination tests. The Caco-2 colorectal carcinoma cells showed equivalent cytotoxic effects from pure CoQ10, which had an IC_50_ value of 8.25 ± 0.38 µg mL^−1^, and ZnO-Q10NPs, which had an IC_50_ value of 8.68 ± 0.27 µg mL^−1^. The cell line showed strong resistance to CuO-Q10NPs, which displayed an IC_50_ value of 46.18 ± 1.36 µg mL^−1^ that was more than five times less effective than pure CoQ10. The reference drugs showed strong effectiveness because Doxorubicin had the lowest IC_50_ value of all tested combinations in this study at 1.01 ± 0.05 µg mL^−1^, while Taxol demonstrated substantial activity with an IC_50_ value of 5.82 ± 0.17 µg mL^−1^. The present study demonstrates that nanoparticle-mediated delivery of CoQ10 significantly modulates its cytotoxic activity in a cell-line-dependent manner. Pure CoQ10 is a highly hydrophobic molecule with poor aqueous solubility (approximately 4 ng mL^−1^), which substantially limits its membrane permeability and intracellular bioavailability at therapeutic concentrations.^39^ Encapsulation into metal oxide nanoparticles is expected to overcome these limitations by improving dispersibility and enabling endocytosis-mediated cellular uptake, thereby increasing the effective intracellular concentration of CoQ10.^40^ The MCF-7 cell experiments show that ZnO-Q10NPs perform much better than free CoQ10 and the two reference drugs, which proves that ZnO-NPs have established pro-oxidant cytotoxicity.

##### 2.2.1.2. Cell cycle analysis

**Fig. 9:**
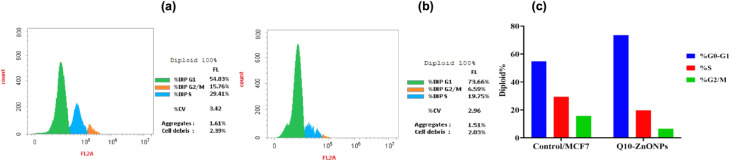
Flow cytometry analysis of cell cycle distribution. (a) Graph of cell cycle analysis against the MCF-7 cell line control. (b) Cell cycle distribution after ZnO-Q10 NPs treatment against the MCF-7 cell line at its IC_50_ (µg mL^−1^). (c) Representative diagram for flow cytometry analysis.

As the lowest IC_50_ value was obtained for ZnO-Q10NPs against the breast cancer cell line, it was subjected to cell cycle analysis. Flow cytometric analysis was conducted to elucidate the mechanism underlying the potent cytotoxic effects of ZnO-Q10 nanoparticles (ZnO-Q10NPs) on the MCF-7 breast cancer cell line. The percentage of MCF-7 cells arrested in the G_0_/G_1_ phase increased significantly to 73.66% compared to 54.83% in untreated control cells (Fig. 9). In contrast, there was a pronounced decrease in cell populations in the S phase (from 29.41% in control to 19.75% after treatment) and the G_2_/M phase (from 15.76% to 6.59%). These findings indicate that ZnO-Q10NPs induce cell cycle arrest predominantly at the G_0_/G_1_ checkpoint. This G_0_/G_1_ arrest disrupts the normal course of the cell cycle, inhibiting the transition into the DNA synthesis (S) phase and the following mitosis (G2/M), in accordance with.^41^ The accumulation of cells in the G_0_/G_1_ phase is associated with increased apoptosis, suggesting that ZnO-Q10NPs not only inhibit proliferation but also actively promote programmed cell death. The significant decline in S and G2/M phase cells further supports the hypothesis that these nanoparticles effectively block cell cycle advancement through synergistic ZnO-mediated lysosomal destabilization, Zn^2+^ release, and ROS generation, coupled with CoQ10's modulation of mitochondrial electron transport and redox imbalance that exacerbates oxidative stress and energy depletion. This mechanism is critical to the nanoparticles' anticancer activity, as halting the cell cycle at G_0_/G_1_ prevents cancer cell replication and growth.

##### 2.2.1.3. Apoptosis detection assay

**Fig. 10:**
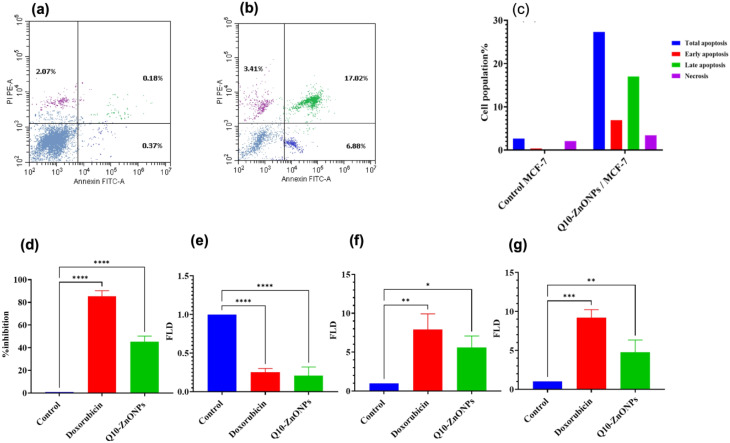
(a) Representative flow cytometer dot plots of MCF-7 cells, (b) ZnO-Q10NPs-induced MCF-7 cell line apoptosis and necrosis %, (c) quantitative analysis of apoptotic cell populations in control and ZnO-Q10NP-treated MCF-7 cells. (d) effect of ZnO-Q10NPs on topoisomerase IIβ, (e) effect of ZnO-Q10NPs on Bcl-2, (f) effect of ZnO-Q10NPs on caspase-3, and (g) effect of ZnO-Q10NPs on Bax. Statistical analysis was conducted using one-way ANOVA followed by a post hoc test.

Flow cytometric analysis using Annexin V-FITC/PI staining, Fig. 10 (a and b), displayed a substantial increase in apoptosis in MCF-7 breast cancer cells after 48 h treatment with ZnO-Q10NPs at the IC_50_ dose compared with untreated controls. Total apoptotic cells rose dramatically, driven by a 6.88-fold increase in early apoptosis and a 17.02-fold increase in late apoptosis, with minimal necrosis observed. These findings indicate that ZnO-Q10NPs predominantly trigger regulated programmed cell death over nonspecific necrosis, contributing to the observed cytotoxicity and cell cycle arrest.^42^ To further elucidate the mechanisms driving this effect, topoisomerase IIβ expression was assessed in Fig. 10(d). ZnO-Q10NPs inhibited topoisomerase IIβ by 45.7% (*p* < 0.0001), disrupting DNA topology, replication, and transcription.^43^ Gene expression profiling Fig. 10(g) revealed Bax upregulation (4.76-fold, *p* < 0.001), Caspase-3 elevation (5.61-fold, *p* < 0.01), Fig. 10(f), and Bcl-2 downregulation (0.21-fold, *p* < 0.00001), Fig. 10(e). The pronounced Bax/Bcl-2 ratio shift supports mitochondrial intrinsic apoptosis activation, where Zn^2+^ ions from ZnO dissolution permeabilize lysosomal membranes, releasing cathepsins that amplify Caspase-3 cleavage, while CoQ10 disrupts electron transport chain complexes I/II, enabling Bax pore formation against Bcl-2 inhibition.^44–46^ This agrees with^47^ ZnO-Q10NPs, which thus exhibit potent pro-apoptotic and anti-proliferative effects through synergistic ROS overproduction, mitochondrial sabotage (CoQ10 redox interference), and DNA process blockade.

##### 2.2.1.4. ZnO-Q10 NPs as a bioactive platform for NRF-2 modulation in anticancer strategies

**Fig. 11:**
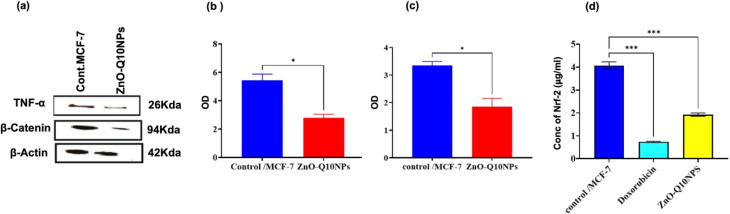
Modulatory effect of ZnO-Q10NPs on protein expression levels in MCF-7 cells. (a) Immunoblot analysis of TNF-α (26 kDa) and β-catenin (94 kDa) expressions, with β-actin (42 kDa) as the loading control. (b and c) Relative densitometric quantification of (b) β-catenin and (c) TNF-α levels normalized to β-actin. (d) ELISA-based quantification of Nrf-2 concentration (µg mL^−1^). Data are expressed as mean ± SD(*n* = 3). Statistical analysis was performed using oneway ANOVA followed by a suitable post hoc test. *p < 0.05, **p.

NRF-2 is a critical regulator of antioxidant responses. In cancer cells, it is usually hyperactive, impeding redox balance and aiding cells to survive oxidative injury.^48^ Along with the substantial elevation of Caspase-3 gene expression as measured by RT-PCR, we also measured the amount of NRF-2 protein by ELISA to evaluate the fine balance of cell death and survival. These two markers were selected for analysis because of their opposing functions in the MCF-7 cellular environment; Caspase-3 is the ultimate effector of apoptosis, whereas NRF-2 is the chief controller of the antioxidant defense that normally shields cancer cells from therapy-induced stress. In the present investigation, ZnO-Q10NPs significantly downregulated the NRF-2 protein expression to 1.925 (*p* < 0.0001) in comparison to the increased baseline levels of 4.068 in the untreated controls^49,50^Fig. 11(d). Interestingly, this lowering of NRF-2 was discovered to be tightly coupled to the already initiated apoptotic signaling. The observed negative correlation shows that ZnO-Q10NP-induced apoptosis is strategically promoted by concurrent inhibition of the NRF-2-mediated antioxidant response. Measuring both indicators, our results demonstrate that the nanoparticles successfully disable the cell's survival system, guaranteeing that the oxidative excess results directly in the activation of the deadly proteolytic cascade. The reference drug, Doxorubicin, was likewise more powerful (0.738) due to its aggressive DNA targeting and generation of ROS; however, ZnO-Q10 NPs provide a more targeted benefit therapeutically. The nanoparticles reduce NRF2 translocation by combining Zn^2+^-induced lysosomal degradation with CoQ10's interference with mitochondria, without the wide toxicity of traditional chemotherapy. The balanced technique sensitizes the tumor to apoptosis efficiently while conserving physiological defense, proposing ZnO-Q10NPs as a feasible targeted breast cancer therapy.^49,51,52^

##### 2.2.1.5. Characterization of ZnO-Q10 NPs effects on TNF-α and β-catenin expression *via* western blot

Building on the fact that ZnO-Q10NPs cause NRF-2 suppression, which throws off redox homeostasis, western blot analysis looked at how TNF-α (a pro-inflammatory cytokine) and β-catenin (a Wnt effector) were suppressed in MCF-7 cells at IC_50_ concentrations. These two proteins are important for the survival-inflammation-proliferation network. Western blot analysis demonstrated that ZnO-Q10NPs treatment caused a significant reduction (*p* < 0.01) in TNF-α and β-catenin expression levels in MCF-7 breast cancer cells compared to untreated controls. TNF-α expression decreased from 3.334 in controls to 1.851 in the ZnO-Q10NPs -treated group, Fig. 11(c), while β-catenin levels dropped from 5.451 to 2.786 following nanoparticle exposure, Fig. 11(b). TNF-α, a pro-inflammatory cytokine, fuels tumor progression by fostering angiogenesis, immune evasion, and resistance to apoptosis within the tumor microenvironment. Elevated TNF-α correlates with breast cancer aggressiveness and poor prognosis.^53^ ZnO-Q10NPs suppress TNF-α through CoQ10's antioxidant scavenging of NF-κB pathway activators combined with ZnO-mediated lysosomal disruption that attenuates inflammatory cytokine secretion, thereby dismantling the pro-tumorigenic inflammatory niche.^54^ β-Catenin serves as a central effector in the canonical Wnt signaling pathway, driving transcription of genes that promote cellular proliferation, survival, and metastasis in breast cancer. Its aberrant stabilization fosters oncogenic signaling and therapeutic resistance. The substantial downregulation by ZnO-Q10NPs arises from ZnO-induced ROS generation that activates GSK-3β kinase, enhancing β-catenin phosphorylation and subsequent proteasomal degradation, thereby disrupting proliferative signaling cascades.^55^ Collectively, these findings demonstrate that ZnO-Q10NPs coordinately target Wnt/β-catenin proliferative signaling and TNF-α inflammatory pathways, revealing their multifaceted anticancer mechanism through synergistic nanoparticle-enhanced delivery and redox modulation.^24,56–58^

After the encouraging *in vitro* results, ZnO-Q10NPs would start a pathway for apoptosis that targets more than one cell type in MCF-7 cells. A significant alteration in the Bax/Bcl-2 ratio, the activation of Caspase-3, and the inhibition of nuclear factors such as β-catenin, TNF-α, and Nrf-2 activity all corroborate this. Stopping topoisomerase II was also highly critical for stopping the cell cycle at G_0_/G_1_ and slowing down how quickly DNA copies itself (Fig. S3). The investigation was expanded to include the Ehrlich Ascites Carcinoma (EAC) model *in vivo*. This was done not only to validate systemic effectiveness but also to make a comparison. The goal of this study was to see how well the produced ZnO-Q10 NPs worked as a treatment compared to both the pure CoQ10 and the CuO-Q10NPs. This kind of comparison gives a clear, evidence-based reason for choosing the best nano-formulation that lowers tumors the most while being the safest.

#### 2.2.2. *In vivo* studies

##### 2.2.2.1. Acute toxicity assessment of the test compounds

CoQ10, ZnO-Q10NPs, and CuO-Q10NPs were administered intraperitoneally to assess acute toxicity and calculate the median lethal dose of CoQ10, ZnO-Q10NPs, and CuO-Q10NPs. Up to 100 mg kg^−1^ of CoQ10, ZnO-Q10NPs, and CuO-Q10NPs were determined to be safe, with no mortality noted, according to the results.^59^

##### 2.2.2.2. Effect of treatment on EAC cell count and volume

**Fig. 12:**
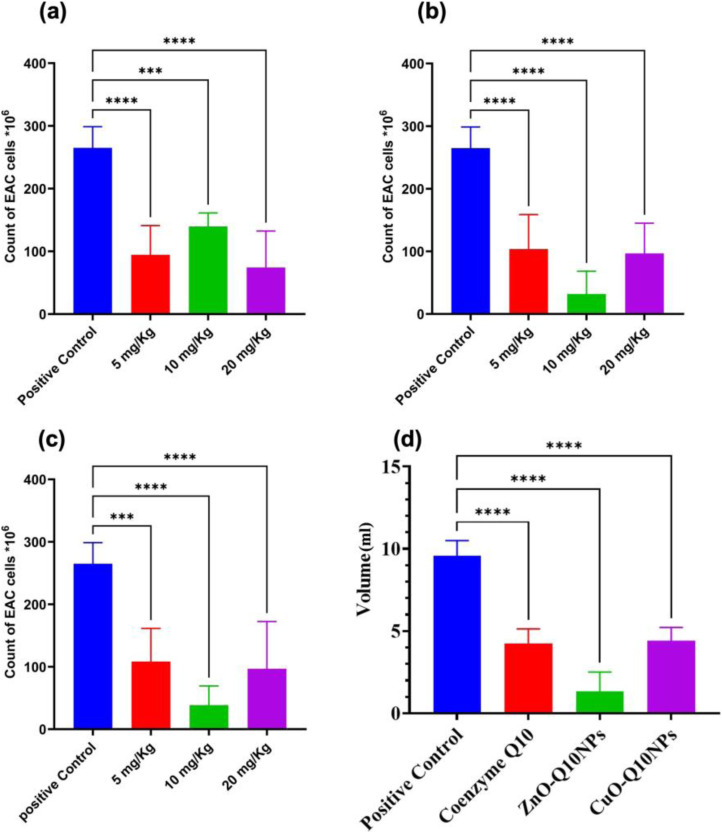
Antiproliferative efficacy of different treatment formulations on EAC tumor burden. Dose–response screening based on total viable EAC cell counts × 106 following administration of (a) pure CoQ10, (b) ZnO-Q10NPs, and (c) CuO-Q10NPs at escalating dosages of 5, 10, and 20 mg kg^−1^. (d) Comparative evaluation of EAC tumor fluid volumes (mL) measured at the selected optimized therapeutic dosages (20 mg kg^−1^ for free CoQ10 and 10 mg kg^−1^ for both nanoformulations). Data are presented as mean ± (SD) (*n* = 6) per group. Statistical analysis was performed using one-way ANOVA followed by a post hoc test. *p < 0.05, **p.

Administration of CoQ10 and its ZnO-Q10NPs and CuO-Q10NPs formulations induced statistically significant reductions in Ehrlich ascites carcinoma (EAC) cell counts and tumor volumes compared to the positive control (*p* < 0.0001), as detailed in Fig. 12. Analysis demonstrated that all treatment groups exhibited a significant decline in EAC cells compared with the positive control (*p* < 0.0001 for all doses), substantiating the antitumor potential of these agents against EAC growth. The results shown in (Fig. 12) indicated that CoQ10 itself and its ZnO- and CuO-nanoparticle forms both inhibited tumor cell growth in a dose-dependent manner. For the CoQ10 group, increasing the dose from 5 to 20 mg kg^−1^ progressively decreased EAC cell counts, with reductions of 64.34% at 5 mg kg^−1^, 47.36% at 10 mg kg^−1^, and 71.92% at 20 mg kg^−1^, Fig. 12(a). ZnO-Q10NPs produced a stronger effect, suppressing EAC cells by 60.88% at 5 mg kg^−1^, 87.99% at 10 mg kg^−1^, and 63.55% at 20 mg kg^−1^. Enhanced efficacy results from ZnO conjugation, improving CoQ10's poor aqueous solubility, enabling receptor-mediated endocytosis, EPR-effect tumor targeting, and Zn^2+^-induced ROS generation that synergizes with CoQ10's mechanisms Fig. 12(b). The CuO-Q10NPs group showed high efficacy, with 59.18% reduction at 5 mg kg^−1^, 85.50% at 10 mg kg^−1^, and 63.52% at 20 mg kg^−1^. Superiority at lower doses stems from the potent cytotoxicity of CuO *via* Cu^2+^ ion release, excessive ROS production, and membrane disruption, amplifying CoQ10's apoptotic effects, though slightly less than ZnO-NPs overall, Fig. 12(c). Among all formulations, ZnO-Q10NPs proved superior, achieving the highest inhibition (87.99% at 10 mg kg^−1^) due to optimal synergy between the biocompatibility of ZnO, sustained ROS modulation, and enhanced CoQ10 bioavailability without the potential toxicity risks of CuO at higher doses. Comparatively, nanoparticle forms outperformed pure CoQ10 at matched doses, but escalating to 20 mg kg^−1^ often yielded diminished returns, suggesting saturation or mild toxicity common in nano-delivery systems. Based on the cell count calculations, the optimal therapeutic doses were determined to be 10 mg kg^−1^ for both nanoformulations (ZnO-Q10NPs and CuO-Q10NPs) and 20 mg kg^−1^ for the pure CoQ10 compound. Consequently, the subsequent measurement of tumor volume was selectively performed using these optimized, highly effective dosages, as detailed in Fig. 12(d). Regarding the tumor volume measurements at these optimized dosages, the untreated EAC control group exhibited a high tumor volume of (9.58 ± 0.92) mL. Pure CoQ10 monotherapy (at 20 mg kg^−1^) significantly reduced the tumor volume to 4.25 ± 0.88 mL (55.65% reduction). Interestingly, the nanoformulations at a lower dose (10 mg kg^−1^) demonstrated a remarkably enhanced antiproliferative efficacy. The ZnO-Q10NPs group achieved the most prominent therapeutic outcome, suppressing the tumor volume by 1.33 ± 1.17 mL (86.12% reduction). Meanwhile, the CuO-Q10NPs formulation decreased the tumor volume to (4.42 ± 0.80) mL, which corresponds to a 53.86% reduction.

##### 2.2.2.3. Hepatorenal protective effects of coenzyme Q10 and its nanoparticle formulations in an EAC model

**Fig. 13:**
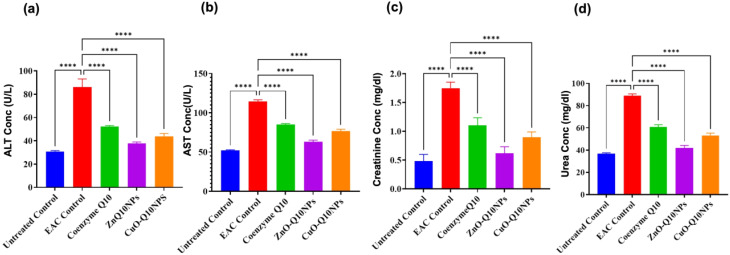
Effect of treatment on (a) ALT (U L^−1^), (b) AST (U L^−1^), (c) creatinine (mg dL^−1^), and (d) urea (mg dL^−1^). Statistical analysis was performed using one-way ANOVA, followed by a post hoc test.*p < 0.05, **p < 0.01, ***p < 0.001, and ****p < 0.0001 indicate significant differences compared to the EAC control.

Fig. 13 serum biomarkers of hepatic and renal functions were measured to assess the hepatorenal protective efficacy of the synthesized nanotherapeutics against EAC-induced systemic toxicity. The combination of free CoQ10 (20 mg kg^−1^) with its nano formulated counterparts, ZnO-Q10NPs (10 mg kg^−1^) and CuO-Q10NPs (10 mg kg^−1^) induced profound modulating effects on the biochemical profiles of Ehrlich ascites carcinoma (EAC) bearing mice as shown in Fig. 13. Specifically, the treatment strategies markedly attenuated hepatic injury as demonstrated by the normalization of serum transaminase activities including alanine aminotransferase (ALT) and aspartate aminotransferase (AST). At the same time, the functional integrity of the kidney was significantly preserved as evidenced by a significant decrease in the levels of serum creatinine and urea when compared to the EAC group. The analysis of serum liver function markers revealed significant alterations in the enzymatic activities of AST and ALT among the different experimental groups, reflecting the hepatic impact of EAC progression and subsequent treatments. As shown in the relevant Fig. 13(a and b), the untreated normal control group-maintained baseline ALT (30.83 ± 0.75 U L^−1^) and AST (52.17 ± 0.75 U L^−1^) levels, consistent with normal liver physiology. In contrast, the EAC control group exhibited a substantial elevation in these enzymes, with ALT reaching 86 ± 7.09 U L^−1^ and AST increasing to 114.67 ± 1.82 U L^−1^ (*p* < 0.00001), signifying severe hepatocellular injury. This elevation indicates leakage of intracellular enzymes into the bloodstream due to EAC-induced hepatic damage or oxidative stress-related membrane disruption.^60^ The significant rise in liver enzyme levels in EAC-bearing mice aligns with previous reports that tumor growth and associated metabolic stress can compromise liver integrity, leading to elevated transaminase activity. Such changes reflect not only direct hepatic impairment but also systemic disturbances caused by tumor metabolism and inflammation. These findings establish a baseline for assessing the protective effects of CoQ10 and its nanoparticle formulations, which will be discussed in subsequent sections focusing on their hepatoprotective potential.^61^ A statistical study demonstrates a significant difference in liver function between the EAC Control and Untreated Control groups (*P* < 0.0001). Treatment with CoQ10 markedly improved liver function parameters compared with the EAC control group. The CoQ10-treated mice exhibited a noticeable decrease in serum ALT to 52.33 ± 0.82 U L^−1^ and AST to 85.17 ± 1.17 U L^−1^, indicating partial hepatoprotection and mitigation of EAC-induced liver injury.^62–64^ This reduction suggests that CoQ10alleviates hepatic damage through its antioxidant properties and ability to stabilize mitochondrial membranes. Further improvements were observed in groups treated with nanoparticle formulations. The coenzyme ZnO-Q10NPs group showed the most significant reductions, with ALT levels decreasing to 37.83 ± 1.17 U L^−1^ and AST to 63.17 ± 2.04 U L^−1^. Similarly, the CuO-Q10NPs group demonstrated considerable decreases in ALT and AST to 43.83 ± 2.48 U L^−1^ and 76.83 ± 2.23 U L^−1^, respectively.^65,66^ Statistical analysis confirmed these reductions were highly significant (*p* < 0.0001) compared with the EAC control. Statistical analysis confirmed these reductions were highly significant (*p* < 0.0001) compared with the EAC control.^66–68^ Notably, the coenzyme ZnO-Q10NPs group's ALT and AST levels approached those of the untreated control group (ALT: 30.83 ± 0.75 U L^−1^; AST: 52.17 ± 0.75 U L^−1^), suggesting a strong ameliorative effect. This enhanced hepatoprotective action can be attributed to the improved bioavailability and synergistic antioxidant effects provided by zinc oxide nanoparticle delivery.

The kidney function evaluation, based on serum creatinine and urea levels, demonstrated significant renal impairment associated with EAC progression Fig. 13(c and d). The negative control group presented with normal baseline creatinine (0.483 ± 0.12 mg dL^−1^) and urea (43.833 ± 1.414 mg dL^−1^) levels, consistent with normal renal function. In contrast, the EAC positive control group showed markedly elevated creatinine (1.75 ± 0.105 mg dL^−1^) and urea (88.83 ± 1.72 mg dL^−1^), reflecting significant renal dysfunction likely due to tumor-induced metabolic derangements or direct renal damage.^69^ The statistical difference between the EAC Control and Untreated Control groups was highly significant (*p* < 0.0001), confirming the degree of kidney injury in this model. Treatment with coenzyme Q10 led to intermediate creatinine (1.105 ± 0.13 mg dL^−1^) and urea (60.83 ± 1.94 mg dL^−1^) values, indicating partial renal protection or altered metabolic handling of these biomarkers compared to untreated EAC controls. This may suggest mild nephrotoxic effects or metabolic modulation related to CoQ10administration.^70,71^

Nanoparticle formulations of CoQ10 further improved renal function markers. The ZnO-Q10NPs group showed creatinine and urea levels (0.617 ± 0.117 mg dL^−1^ and 42 ± 2.191 mg dL^−1^, respectively) closest to normal controls, indicating strong reno-protective effects. The CuO-Q10NPs group exhibited mild to moderate elevations (creatinine 0.9 ± 0.089 mg dL^−1^; urea 53.167 ± 2.137 mg dL^−1^), suggesting renal benefits, albeit less pronounced than the zinc nanoparticle formulation.^72–74^ The reductions in creatinine and urea compared to the EAC control were statistically significant (*p* < 0.0001), highlighting the therapeutic potential for mitigating EAC-associated nephrotoxicity.^75^ Differences in the efficacy of ZnO-NPs and CuO-NPs formulations may be due to their distinct physicochemical properties and interactions with CoQ10. Zinc's well-known antioxidant and anti-inflammatory properties likely synergize with CoQ10 to provide enhanced renal protection, while copper's potential pro-oxidant effects at elevated concentrations may limit its efficacy. These findings underscore the advantage of zinc oxide nanoparticle conjugation in enhancing CoQ10's therapeutic effects in maintaining kidney function amid cancer-related renal impairment.

##### 2.2.2.4. Coenzyme Q10 and nanoparticle mediated amelioration of cancer associated lipid dysregulation

**Fig. 14:**
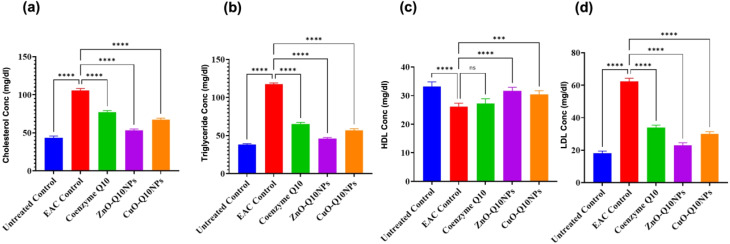
Effect of Treatments on (a) cholesterol conc (mg dL^−1^), (b) triglyceride conc (mg dL^−1^), (c) HDL conc (mg dL^−1^), and (d) LDL conc (mg dL^−1^). Statistical analysis was performed using one-way ANOVA, followed by a post hoc test.*p < 0.05, **p < 0.01, ***p < 0.001, and ****p < 0.0001 indicate significant differences compared to the EAC control.

The serum lipid profile revealed pronounced dyslipidemia in the EAC control group, marked by significantly elevated total cholesterol(107.67 ± 2.58 mg dL^−1^) and triglycerides (117.33 ± 1.97 mg dL^−1^), alongside reduced HDL (26.17 ± 1.17 mg dL^−1^) and elevated LDL (62.33 ± 1.97 mg dL^−1^), compared to healthy controls (total cholesterol 43.3 ± 2.42 mg dL^−1^, triglycerides 38.33 ± 1.03 mg dL^−1^, HDL 33.17 ± 1.60 mg dL^−1^, LDL 43.83 ± 1.41 mg dL^−1^; *P* < 0.0001, Fig. 14). These alterations reflect tumor-induced oxidative stress, which disrupts lipid homeostasis, fosters inflammation, and drives oncogenesis findings consistent with established links between dyslipidemia and cancer progression. CoQ10 administration partially ameliorated these imbalances, reducing total cholesterol to 77.17 ± 2.04 mg dL^−1^ and triglycerides to 65 ± 2.28 mg dL^−1^, while modestly elevating HDL to27.17 ± 1.72 mg dL^−1^ and lowering LDL to34 ± 1.41 mg dL^−1^*P* < 0.0001). This modulation underscores CoQ10's multifaceted role as a mitochondrial bioenergetic cofactor and potent lipophilic antioxidant, which curbs lipid peroxidation and restores enzymatic regulation of lipid metabolism.^76,77^ Notably, CoQ10-loaded ZnO nanoparticles (ZnO-Q10NPs) exhibited superior efficacy, normalizing total cholesterol (53.17 ± 1.94 mg dL^−1^) and triglycerides (46 ± 1.41 mg dL^−1^) to near-control levels, boosting HDL to 31.67 ± 1.21 mg dL^−1^, and markedly reducing LDL to 23 ± 1.55 mg dL^−1^ (*P* < 0.0001). CuO-Q10NPs also improved profiles (total cholesterol 67.5 ± 1.76/dL, triglycerides 56.67 ± 2.23 mg dL^−1^, HDL 30.33 ± 1.37 mg dL^−1^, LDL 30 ± 1.41 mg dL^−1^, *P* < 0.0001), Though less potently.^78^ The enhanced performance of nanoparticle formulations, particularly CoQ10-ZnONPs, likely stems from optimized CoQ10 bioavailability through nanoscale encapsulation, which improves solubility, stability, and targeted cellular uptake. Synergistically, the intrinsic antioxidant and anti-inflammatory properties of ZnO amplify lipid-regulating effects, countering oxidative dysregulation more effectively than free CoQ10 or CuO-Q10NPs, where copper's pro-oxidant potential at elevated doses may limit benefits. Collectively, these data position CoQ10 nanotherapeutics, especially ZnO-based systems, as promising adjuvants for mitigating cancer-associated dyslipidemia and metabolic reprogramming in tumorigenesis.^79–81^

##### 2.2.2.5. Modulation of apoptotic, proliferative, and inflammatory biomarkers in liver tissue by coenzyme Q10 and its nanoparticles using real-time PCR

**Fig. 15:**
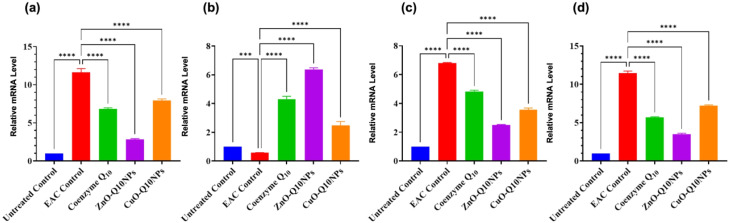
Effect of Treatments on target gene expression levels measured by RT-qPCR. (a) TOPOII, (b) Bax, (c) B-catenin, (d) IL-1B. Statistical analysis was performed using one-way ANOVA, followed by a post hoc test.*p < 0.05, **p < 0.01, ***p < 0.001, and ****p < 0.0001 indicate significant differences compared to the EAC control.

In EAC-bearing controls, liver tissue exhibited marked upregulation of TOPO II (11.61 ± 0.51) and IL-1 (11.46 ± 0.27), alongside elevated β-Catenin (3.57 ± 0.11) and suppressed Bax (0.58 ± 0.02), *versus* untreated controls (*p* < 0.0001; Fig. 15). These shifts signify heightened proliferation, inflammation, β-Catenin-driven oncogenesis, and apoptosis evasion—hallmarks of tumor-induced hepatic dysregulation consistent with IL-1's role in tumor microenvironments and TOPO II's link to genomic instability.^82^ The effects of different CoQ10 treatments on liver tissue biomarkers reveal a nuanced interplay between apoptosis, inflammation, and cell proliferation in the context of EAC. The CoQ10drug control group demonstrated partial biomarker normalization, with reductions in TOPO II (6.82 ± 0.16) and IL-1 (5.69 ± 0.08), alongside increased Bax (4.31 ± 0.19) and a moderate reduction in β-Catenin (4.82 ± 0.09), indicating some restoration of apoptotic function and a modest decrease in oncogenic proliferation. Statistical analysis (*p* < 0.0001).^83,84^ ZnO-Q10NPs demonstrated superior efficacy, sharply decreasing TOPO II (2.85 ± 0.09) and IL-1 (3.52 ± 0.11), maximizing Bax induction (6.38 ± 0.11), and minimizing β-Catenin (2.50 ± 0.03; *p* < 0.0001). In contrast, CuO-Q10 NPs yielded inferior outcomes, with residual TOPO II (7.93 ± 0.20), IL-1 (7.23 ± 0.09), modest Bax (2.48 ± 0.27), and elevated β-Catenin (6.80 ± 0.04). The enhanced performance of ZnO nanoparticles likely stems from improved CoQ10 bioavailability, pH-responsive release, and Zn-mediated Nrf2 activation, suppressing Wnt/proliferation pathways, while the prooxidant potential of CuO nanoparticles at tumor-relevant doses limits efficacy. This agrees with the *in vitro* study. These findings position ZnO-Q10NPs as potent adjuvants targeting apoptosis resistance and inflammatory oncogenesis in EAC.^84–86^

##### 2.2.2.6. Comparative modulation of antioxidant and oxidant biomarkers by coenzyme Q10 and its nanoparticles in liver and kidney tissues

**Fig. 16:**
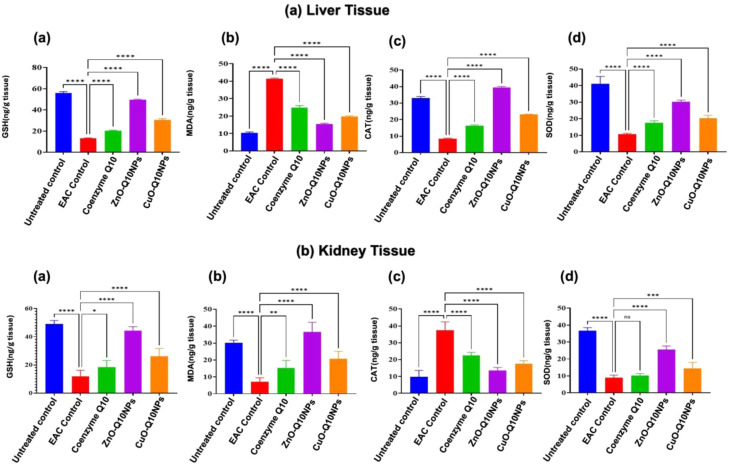
Effect of CoQ10, ZnO-Q10NPs, and CuO-Q10NPs treatments on oxidative stress and antioxidant biomarkers. The evaluation was performed in (a) liver tissue and (b) kidney tissue across the experimental cohorts, representing the changes in (a) GSH (ng g^−1^ tissue), (b) MDA (ng g^−1^ tissue) content as a lipid peroxidation marker, (c) CAT(ng g^−1^ tissue), and (d) SOD (ng g^−1^ tissue). All values are expressed as mean ± (SD) (*n* = 6) per group. Statistical analysis was performed using one-way ANOVA, followed by a post hoc test.*p < 0.05, **p < 0.01, ***p < 0.001, and ****p < 0.0001 indicate significant differences compared to the positive control.

EAC induction profoundly disrupted hepatic antioxidants, depleting GSH (13.10 ± 0.51 nmol mg^−1^), CAT (8.35 ± 0.42 U mg^−1^), and SOD (10.63 ± 0.55 U mg^−1^) while elevating MDA (41.47 ± 0.35 nmol mg^−1^) *versus* untreated controls (GSH 56.03 ± 1.21, CAT 32.93 ± 1.08, SOD 41.20 ± 4.32, MDA 10.33 ± 0.52; *p* < 0.0001; Fig. 16(a)). These shifts reflect tumor-driven ROS overproduction, exhausting enzymatic defenses and driving lipid peroxidation—key mediators of hepatic oncogenesis.^87^ CoQ10 partially restored balance (GSH 20.38 ± 0.43, CAT 16.37 ± 0.39, SOD 11.62 ± 0.46, MDA 25.07 ± 0.39; *p* < 0.0001), attributable to its mitochondrial electron shuttling and free radical scavenging, which curbs ROS propagation.^88^ ZnO-Q10NPs excelled, nearly normalizing GSH (49.62 ± 0.49), surpassing control CAT (39.52 ± 0.60), boosting SOD (30.27 ± 1.03), and slashing MDA (15.33 ± 0.52; *p* < 0.0001).CuO-Q10NPs showed intermediate gains (GSH 30.43 ± 1.23, CAT 23.07 ± 0.24, SOD 16.73 ± 0.63, MDA 19.68 ± 0.39), outperforming free CoQ10 but trailing ZnO-NPs, likely due to the superior ZnO nanoparticle-mediated Nrf2 induction and biocompatibility *versus* the redox duality of CuO.^89^ Parallel renal disruptions emerged in EAC controls (GSH 11.80 ± 4.42, CAT 7.15 ± 2.32, SOD 9.02 ± 1.42, MDA 37.50 ± 4.89 nmol mg^−1^) *versus* controls (GSH 49.10 ± 2.46, CAT 30.23 ± 1.53, SOD 36.60 ± 1.92, MDA 9.83 ± 3.76; *p* < 0.0001; Fig. 16(b)), signaling systemic oxidative insult.^90^ CoQ10 partially ameliorated deficits (GSH 20.38 ± 0.43, CAT 16.37 ± 0.39, SOD 17.64 ± 1.10, MDA 24.65 ± 1.33), yet remained suboptimal.^91^ ZnO-Q10NPs optimally recovered parameters (GSH 49.62 ± 0.49, CAT 39.52 ± 0.60, SOD 30.27 ± 1.03, MDA 15.28 ± 0.52; *p* < 0.0001), while CuO-Q10NPs lagged (GSH 30.43 ± 1.23, CAT 23.07 ± 0.24, SOD 20.23 ± 1.89, MDA 19.77 ± 0.39). ZnO-NPs' enhanced tissue penetration and synergistic antioxidant mimicry underscore their superiority in countering EAC-mediated redox imbalance across organs.^92^

##### 2.2.2.7. Histopathological findings

**Fig. 17:**
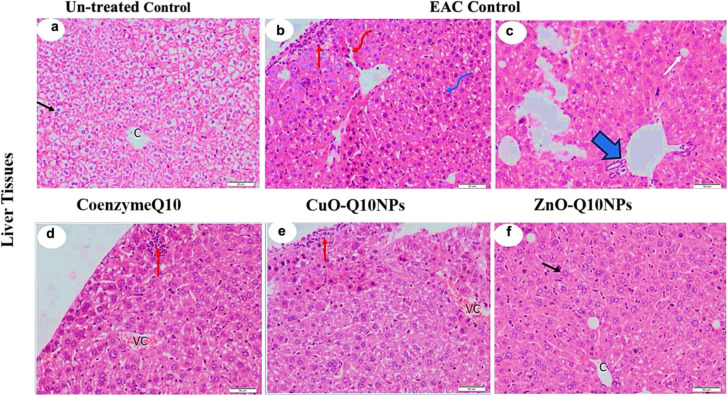
Representative photomicrograph of H&E-stained hepatic tissue sections of control (a), EAC (b and c), CoenzymeQ10 (d), CuO-Q10NPs (e), and ZnO-Q10NPs (f) groups. Notable features include central veins (C), hepatocytes (thin black arrows), malignant Ehrlich ascites carcinoma cells (thin red arrows), Kupffer cell hyperplasia (wavy blue arrow), sinusoidal congestion and dilatation (wavy red arrow), vascular congestion (VC), portal area proliferation (thick blue arrow), vacuolar degeneration (thin white arrow).

**Fig. 18:**
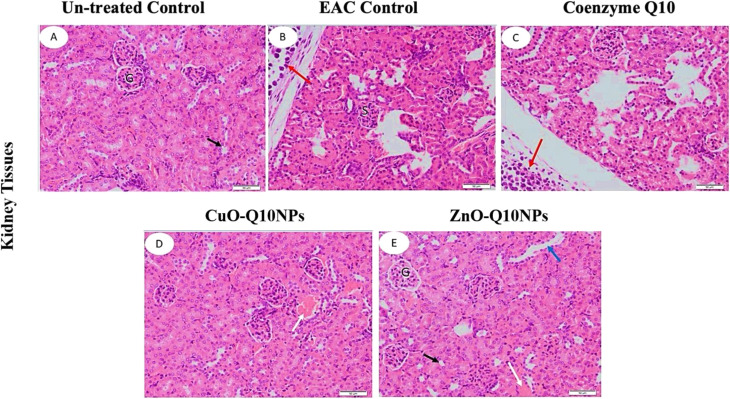
Representative photomicrograph of H&E-stained renal tissue sections of control (A), EAC (B), CoQ10 (C), CuO-Q10NPs (D), and ZnO-Q10NPs (E) groups. Notable features include glomerulus (G), renal tubular series (thin black arrows), massive growth of malignant Ehrlich ascites carcinoma cells (thin red arrows), peritubular vascular congestion (thin white arrows), and dilatation of tubular lumen (thin blue arrows).

**Fig. 19:**
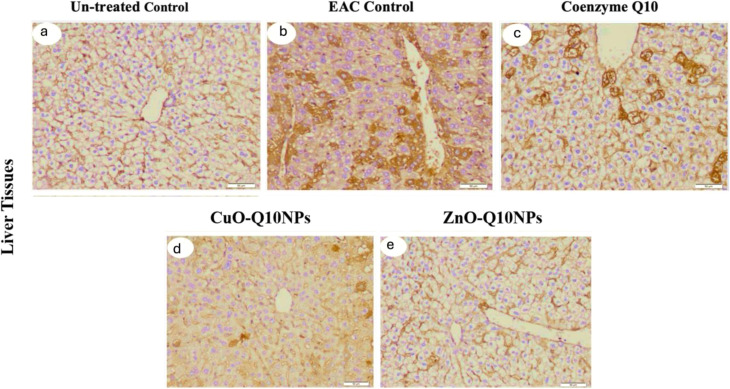
IL6 immunohistochemical expression in hepatic tissues of untreated control (a), EAC (b), CoQ10 (c), CuO-Q10NPs (d), and ZnO-Q10NPs (e) groups. IL6-positive cells are identified by the brown immunoreactive staining. Immunohistochemical staining was counterstained with hematoxylin.

**Fig. 20:**
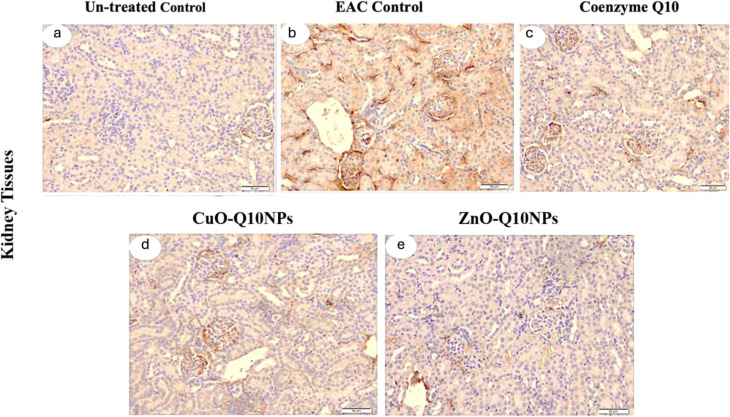
IL6 immunohistochemical expression in renal tissues of untreated control (a), EAC (b), CoQ10 (c), CuO-Q10NPs (d), and ZnO-Q10NPs (e) groups.IL6-positive cells are identified by the brown immunoreactive staining. Immunohistochemical staining was counterstained with hematoxylin.

To further investigate the *in vivo* efficacy of CoQ10, ZnO-Q10NPs, and CuO-Q10NPs against Ehrlich ascites carcinoma (EAC) cells in a mouse model, hepatic and renal tissue specimens were analyzed at a histopathological and immunohistochemical level. Photomicrographs of hematoxylin and eosin (H&E)-stained hepatic and renal tissue sections obtained from the untreated control, EAC, CoQ10, ZnO-Q10NPs, and CuO-Q10NPs groups are presented in Fig. 17 and 18, respectively. In addition, the immunoreactivity of IL-6 proteins in different tissue sections from the untreated control animals and various experimental groups is shown in Fig. 19 and 20, respectively.

###### 2.2.2.7.1. Histopathological alterations in the hepatic tissues

Examination of H&E-stained hepatic tissue sections, obtained from the untreated control group, displayed normal histoarchitecture. The hepatic lobules were found in the form of centrally located veins that form their central axis. Each vein was surrounded by radiating anastomosing cords of polygonal eosinophilic hepatocytes with round vesicular nuclei and large prominent nucleoli. Additionally, hepatic sinusoids, located between hepatocytic plates, were lined with two types of cells: endothelial and Kupffer cells. Fig. 17(a). EAC-treated animals revealed several histological alterations, which were found in the form of massive tumor growth on the outer surface of the liver and within the hepatic parenchyma. Most of the hepatocytes displayed variable degrees of cytoplasmic vacuolations and pyknotic nuclei. Slightly dilated and congested central vein and hepatic sinusoids, along with Kupffer cells hyperplasia and portal area proliferation, were noticed among this group of Fig. 17(b and c). The hepatic tissues of the CoQ10 and CuO-Q10 NPs groups revealed minimal presence of tumor growth, mild hepatic variations, and lesser vascular congestion when compared to the EAC group, Fig. 17(d and e). Histological examination of hepatic issues of the ZnO-Q10NPS group demonstrated restoration of hepatic parenchymal architecture to almost the normal hepatic picture, Fig. 17(f).

###### 2.2.2.7.2. Histopathological alterations in the renal tissues

Histological evaluation of the kidneys of the untreated control group showed normal histological structure with normal renal corpuscles and different tubular series. The corpuscles were mainly formed of numerous loops of blood capillaries (glomeruli), surrounded by an intact Bowman's capsule. The latter was mainly composed of double epithelial layers: the outer parietal and the inner visceral ones, and the Bowman's space was located in between. Concerning the tubular series, their epithelial lining ranged from cuboidal to columnar in type with prominent vesicular nuclei. Fig. 18(A). On the other hand, EAC-treated rats revealed extensive growth of multilayered EAC cells on the external kidney surface, marked renal histological alterations, including shrunken glomerular structures with wide subcapsular Bowman's spaces, congestion of some glomeruli, and inter-tubular capillaries with the presence of extravasated RBCs. Additionally, sloughed and exfoliated epithelial cells, tubular cell swelling with pyknotic nuclei, along with interstitial inflammatory cell invasions, were also observed in Fig. 18(B). Similarly, the CoQ10-treated animals exhibited the histological picture as the EAC group, Fig. 18(C).

Interestingly, both CuO-Q10NPs and ZnO-Q10NPs groups demonstrated significant improvement with slightly declined degenerative changes compared with the EAC-treated group. Fig. 18(D and E). Restoration of nearly-normal renal architecture was observed within ZnO-Q10NPs-treated animals, similar to the untreated control rats, with the presence of slightly peritubular vascular congestion.

##### 2.2.2.8. Immunohistochemical staining of interleukin-6 on liver and kidney tissues

Immunohistochemistry of hepatic parenchymal tissue revealed different patterns of expression of interleukin-6 (IL-6) in the experimental groups (Fig. 19). The normal control and the ZnO-Q10NPs treated groups showed mild to weak IL-6 immunoreactivity throughout the hepatic architecture Fig. 19(a and e). In contrast, the EAC untreated control group showed severe exacerbation of inflammation with high intensity of IL-6 positive cells, Fig. 19(b). Meanwhile, treatment with either free Coenzyme Q10 or CuO-Q10NPs, respectively, modulated this inflammatory response and induced a mild to moderate IL-6 immunoreactivity in the liver parenchyma, Fig. 19(c and d) IL-6 immunoreactivity was simultaneously assessed in renal parenchymal structures (Fig. 20). The normal control and ZnO-Q10NPs groups showed low inflammatory activity at baseline with weak to mild IL-6 immuno expression (Fig. 20(a and e)). After induction of EAC, the renal tissue of the positive control group demonstrated severe inflammation infiltration as shown by the intense and widespread presentation of IL-6-positive cells, Fig. 20(b). However, in the kidneys of mice treated with either free Coenzyme Q10 or CuO-Q10NPs, a significant reduction in mild-to-moderate IL-6 immunoreactivity was observed, indicating a clear renal protection Fig. 20(b).

##### 2.2.2.9. Statistical evaluation of IL-6 immunohistochemistry as an indicator of inflammation in the liver and kidney

**Fig. 21:**
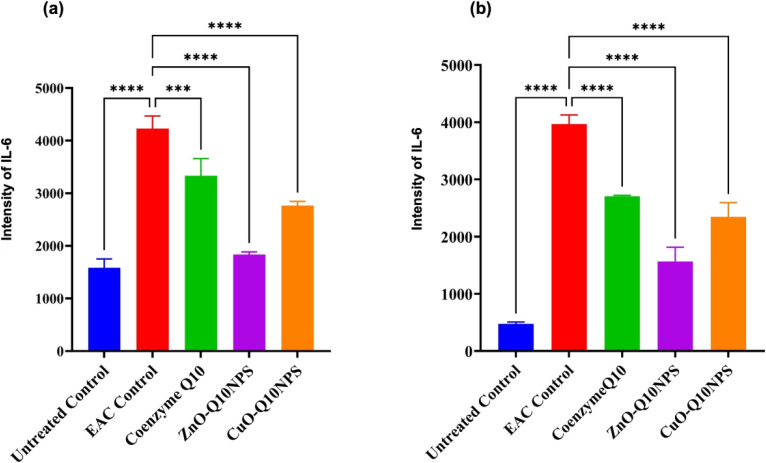
Effect of different treatments on the IL-6 immunohistochemical expression in (a) liver and (b) kidney tissues. Data are expressed as mean intensity of IL-6 immunostaining ± SD for the untreated control, EAC control, CoQ10-treated, ZnO-Q10NPs-treated, and CuO-Q10NPs-treated groups (*n* = 3 per group). The EAC control group showed a marked increase in the IL-6 intensity compared with the untreated control, while treatment with CoQ10, ZnO-Q10NPs, and CuO-Q10NPs significantly reduced IL-6 expression, with the greatest reduction observed in the nanoparticle-treated groups. Statistical analysis was performed using one-way ANOVA, followed by a post hoc test.*p < 0.05, **p < 0.01, ***p < 0.001, and ****p < 0.0001 indicate significant differences compared to the positive control.

Immunostaining analysis of interleukin-6 (IL-6), a key pro-inflammatory cytokine, demonstrated clear stratification across experimental groups, reflecting varying inflammatory activity in liver and kidney tissues (Fig. 21). The untreated control group showed baseline low IL-6 levels (liver: 1583 ± 168.455; kidney: 474.667 ± 32.593), indicative of physiological homeostasis without significant immune activation. Ehrlich Ascites Carcinoma (EAC) induction triggered a marked elevation in IL-6 immunoreactivity (liver: 4229.333 ± 238.548; kidney: 3969 ± 157.344), characteristic of the model's aggressive pathophysiology. This increase arises from tumor-induced hypoxic stress and tumor-derived factors activating pathways in Kupffer cells (liver) and tubular epithelia (kidney), driving cytokine storms that promote fibrosis and tissue injury. CoQ10 treatment produced a moderate IL-6 reduction (liver: 3331.333 ± 329.008; kidney: 2705 ± 15.395) compared to EAC controls, confirming its anti-inflammatory role. CoQ10 acts by scavenging reactive oxygen species (ROS) and enhancing mitochondrial function; however, poor aqueous solubility limits bioavailability, preventing full normalization to control levels. CoQ10 as CuO-Q10NPs achieved greater suppression (liver: 2766.333 ± 80.164; kidney: 2343.667 ± 249.717), outperforming free CoQ10. Nanoencapsulation boosts endocytosis-mediated uptake and site-specific delivery, while Cu^2+^ ions provide additive anti-inflammatory effects through ROS quenching and disruption of tumor microenvironment signaling. CoQ10 as ZnO-Q10NPs yielded the most significant IL-6 downregulation (liver: 1834.667 ± 50.738; kidney: 1565.333 ± 249.171), nearly restoring levels to untreated controls. Zinc's immunomodulation *via* membrane stabilization and upregulation of superoxide dismutase, combined with ZnO-NPs' enhanced permeability/retention and sustained release, optimizes anti-inflammatory efficacy in EAC inflammation.

## 3. Conclusion

In terms of performance and molecular regulation, we observed that ZnO-Q10 NPs work better than CuO-Q10NPs and free CoQ10 for treating breast cancer. CoQ10 was mixed with zinc oxide nanostructures that don't break down readily and are hard for the body to use. We discovered that the potent anticancer properties of ZnO-Q10NPs arise from a mechanism that influences several factors. Changes in the Bax/Bcl-2 apoptotic balance, the activation of Caspase-3, and the stopping of the TNF-α/IL-1β/IL-6 inflammatory cascade back this up. You can stop cancer from growing and keep the redox balance in cells by managing Nrf-2, β-catenin, and topoisomerase. ZnO-Q10 is a very accurate nanomedicine that alters the pH and kills certain cells. It can help treat metastatic breast cancer by lowering the body's overall toxicity and raising the mortality rate.

## Conflicts of interest

The authors declare that they have no conflict of interest.

## Supplementary Material

RA-OLF-D6RA02842J-s001

RA-OLF-D6RA02842J-s002

## Data Availability

The data supporting this study are available from the corresponding author upon reasonable request. Supplementary information (SI): additional figures and tables supporting the main text. See DOI: https://doi.org/10.1039/d6ra02842j.
