# Supplementary material for: Optimized CoQ10 delivery via ZnO/CuO nanoparticles: greater in vitro cell cycle arrest and in vivo tumor suppression than unformulated coenzyme Q10
Source: RSC Adv. 2026 Jul 21. Online ahead of print. doi: 10.1039/d6ra02842j (PMC13386344; doi:10.1039/d6ra02842j)

Supplementary Information (SI) for RSC Advances.
This journal is © The Royal Society of Chemistry 2026

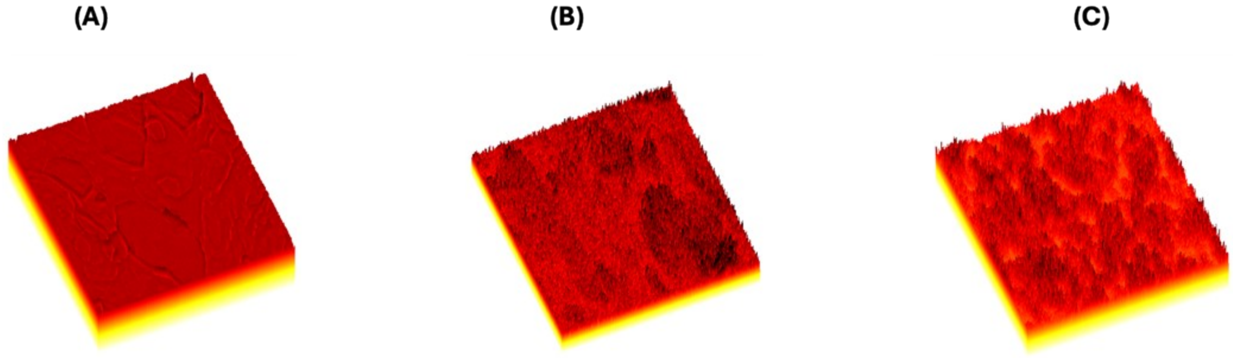

Fig.S1 Roughness micrographs of A) CoQ10, B) ZnO-Q10NPs, and C) CuO-Q10NPs.

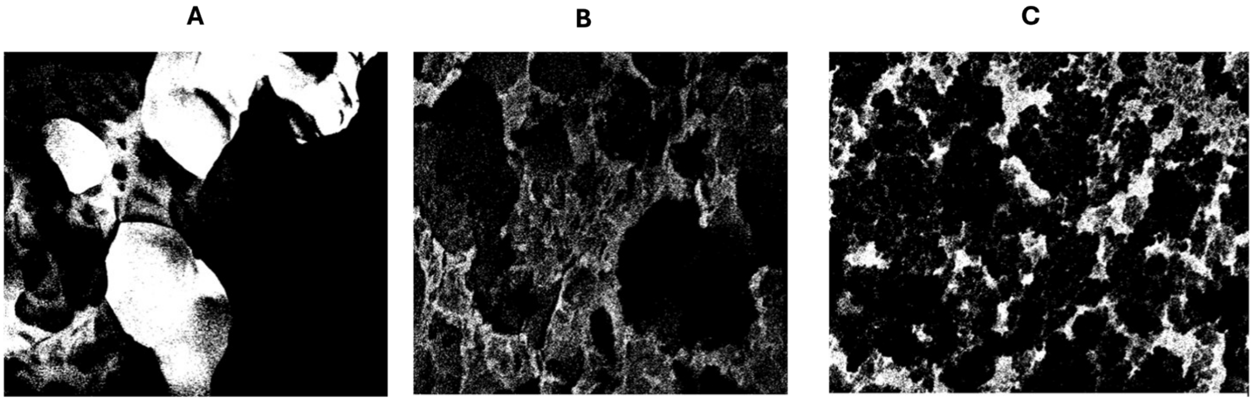

Fig. S2 Porosity micrographs of A) CoQ10, B) ZnO-Q10NPs, and C) CuO-Q10NPs.

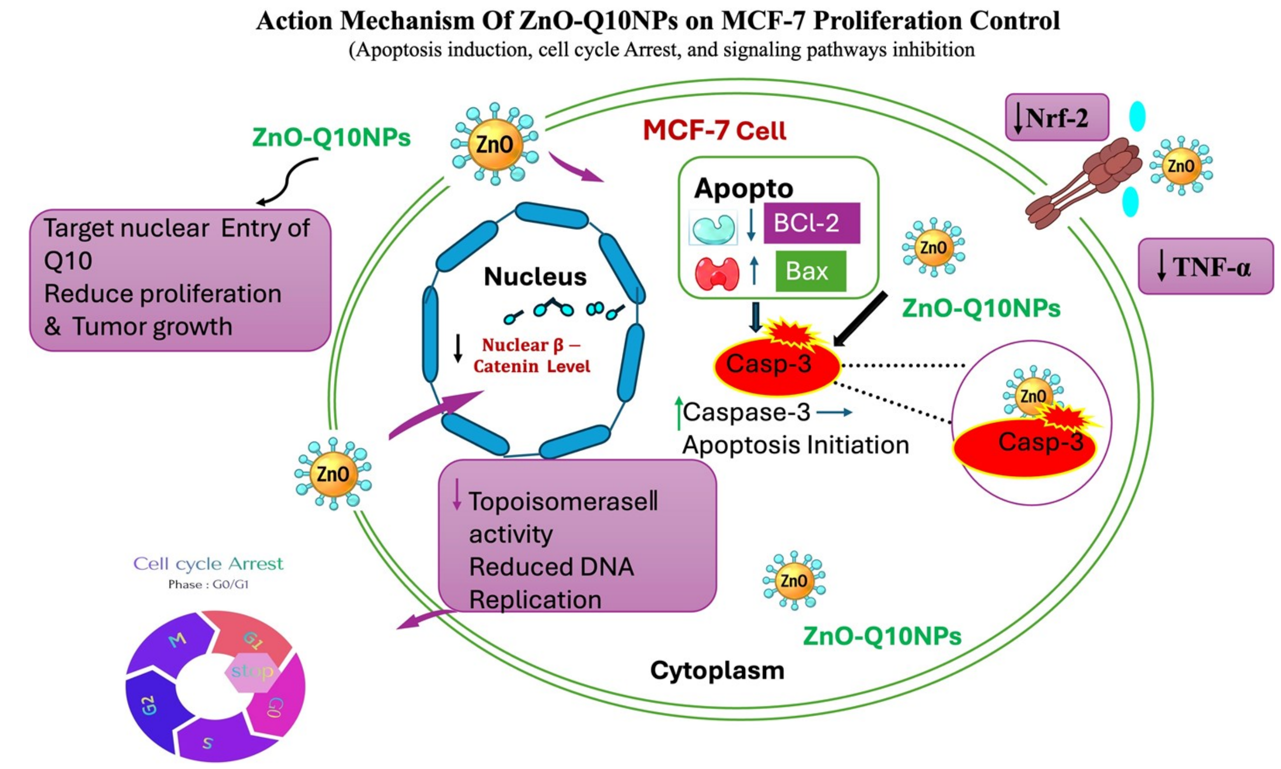

Fig.S 3 Proposed molecular mechanism of ZnO-Q10 NPs anti-tumor activity in MCF-7 cells. The schematic illustrates the multi-targeted approach of the nanoparticles, including the induction of apoptosis via modulation of the Bax/Bcl-2 ratio and activation of the Caspase-3 cascade. It also highlights the inhibition of the β-catenin signaling pathway and Topoisomerase II activity, which leads to G0/G1 cell cycle arrest and suppressed DNA replication. Additionally, the formulation downregulates TNF-α and Nrf-2 pathways, collectively resulting in reduced cell proliferation and inhibited tumor growth.

Lab Report

s	Compound			western blotting		β-actin
				OD		
	code	cells	conc	94 kda	26 kDa	
				B-Catenin	TNFα	
I	ZnO-Q10NPs	MCF7	---	2.013	1.851	√
III	Control MCF-7	---	---	5.451	3.334	√

ZnO-q10NPS Cont.M

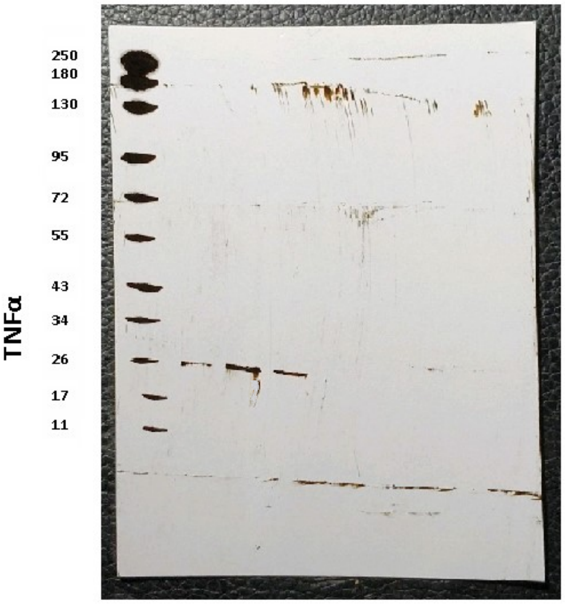

Cont.M ZnO-q1

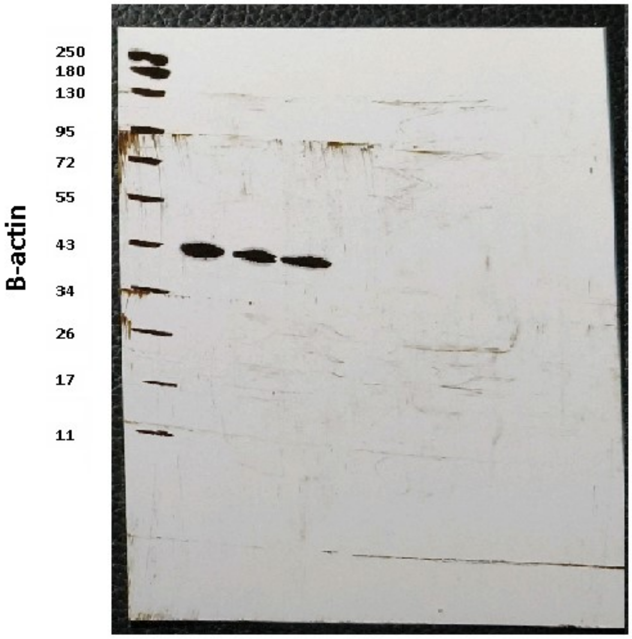

Cont.M ZnO-q1

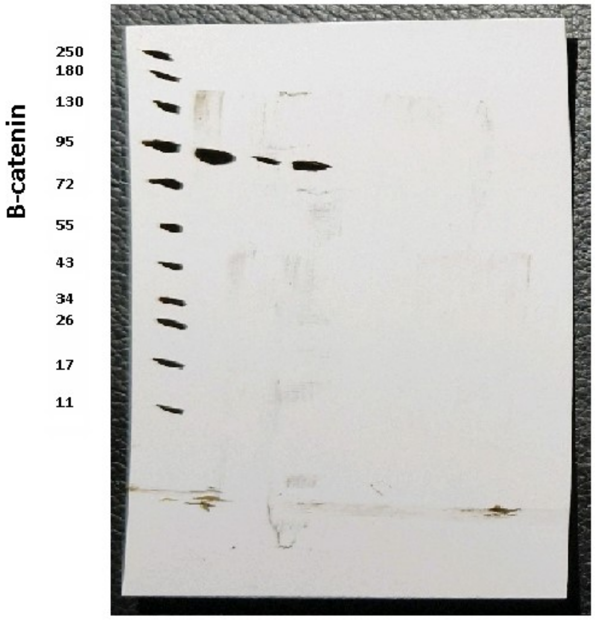

Supplement: RA-OLF-D6RA02842J-s001 [file RA-OLF-D6RA02842J-s001.pdf]
