# Supplementary material for: Optimized CoQ10 delivery via ZnO/CuO nanoparticles: greater in vitro cell cycle arrest and in vivo tumor suppression than unformulated coenzyme Q10
Source: RSC Adv. 2026 Jul 21. Online ahead of print. doi: 10.1039/d6ra02842j (PMC13386344; doi:10.1039/d6ra02842j)
Supplement: RA-OLF-D6RA02842J-s002 [file RA-OLF-D6RA02842J-s002.pdf]

This journal is © The Royal Society of Chemistry 2026

Tables

Table S1: qRT-PCR Analysis primer sequences for apoptosis-related genes and housekeeping control.

	Bax	BCl2	IL1β	β-Catenin	TOPO II	Caspase-3	GAPDH
**Forward**	5'-TCAGGATGCGTCCACCAAGAAG -3'	5'-ATCGCCCTGTGGATGACTGAGT -3'	5'-CCACAGACCTTCCAGGAGAATG -3'	5'-CACAAGCAGAGTGCTGAAGGTG-3'	5'-GTGGCAAGGATTCTGCTAGTCC -	5'-GTTTGAGGACCTTCGACCAGCT-3'	5'-GTCTCCTCTGACTTCAACAGCG -3'
**Reverse**	5'-TGTGTCCACGGCGGCAATCATC -3'	5'-GCCAGGAGAAATCAAACAGAGGC -3'	5'-GTGCAGTTCAGTGATCGTACAGG-3'	5'-GATTCCTGAGAGTCCAAAGACAG-3'	5'-ACCATTCAGGCTCAACACGCTG -	5'-CAACGTACCAGGAGCCACTCTT-3'	5'-ACCACCCTGTTGCTGTAGCCAA -3'

Table S2: Roughness parameters of A) CoQ10, B) ZnO-Q10NPs, and C) CuO-Q10NPs samples.

Roughness parameters	Co-Q10	ZnO-Q10NPs	CuO-Q10NPs.
Root Mean Square Roughness (R_q)	1.17	2.18	3.08
Roughness Average (R_a)	1.37	4.76	9.49
Maximum Average Height (R_z)	13.33	30.83	62.42
Porosity %	24.13%	1.91%	2.67%

Table S3: The elemental analysis of ZnO-Q10 nanocomposites.

ZnO-Q10NPs		
Element	Weight%	Atomic%
C	48.28	66.38
O	26.37	27.22
Zn	25.35	6.41

Table S4: CuO-Q10 nanocomposites.

CuO-Q10NPs.		
Element	Weight%	Atomic%
C	49.72	65.51
O	29.67	29.35
Cu	20.51	5.13

Table S5 shows the effect of the synthesized compounds on the viability of the Mcf-7, Caco2, and HepG-2 cell lines, as measured at different concentrations of the tested samples.

Sample	Cell line	Coenzyme-Q10	ZnO-Q10NPs	CuO-Q10NPs	Doxorubicin	Taxol
IC_{50} ± SEM (μg/ml)	**Mcf-7**	**7.711**±0.24	**3.645**±0.12	**10.49**±0.36	**13.29**±0.41	5.421±0.18
	**HepG2**	**13.31**±0.61	**11.78**±0.35	**9.462**±0.28	**2.303**±0.11	3.524±0.11
	**Caco$_2$**	8.259±0.38	8.687±0.27	46.18±1.36	1.011±0.05	5.822±0.17
